# Overexpressing the ClpC AAA+ unfoldase accelerates developmental cycle progression in *Chlamydia trachomatis*

**DOI:** 10.1128/mbio.02870-24

**Published:** 2024-11-22

**Authors:** Aaron A. Jensen, Saba Firdous, Lei Lei, Derek J. Fisher, Scot P. Ouellette

**Affiliations:** 1 Department of Pathology, Microbiology, and Immunology, College of Medicine, University of Nebraska Medical Center, Omaha, Nebraska, USA; 2 Laboratory of Clinical Immunology and Microbiology, National Institute of Allergy and Infectious Diseases, National Institutes of Health, Bethesda, Maryland, USA; 3 School of Biological Sciences, Southern Illinois University Carbondale, Carbondale, Illinois, USA; University of Maryland School of Medicine, Baltimore, Maryland, USA; University of Maryland, Baltimore, Baltimore, Maryland, USA

**Keywords:** *Chlamydia*, ClpC, ClpP, AAA+ ATPase, Clp protease, differentiation, development

## Abstract

*Chlamydia* is an obligate intracellular bacterium that undergoes a complex biphasic developmental cycle, alternating between the smaller, infectious, non-dividing elementary body (EB) and the larger, non-infectious but dividing reticulate body. Due to the differences between these functionally and morphologically distinct forms, we hypothesize protein degradation is essential to chlamydial differentiation. The bacterial Clp system, consisting of an ATPase unfoldase (e.g., ClpX or ClpC) and a proteolytic component (e.g., ClpP), is critical for the physiology of bacteria through its recognition, and usually degradation, of specific substrates. We observed by transmission electron microscopy that overexpression of wild-type ClpC, but not an ATPase mutant isoform, in *Chlamydia* increased glycogen accumulation within the vacuolar niche of the bacteria earlier in the developmental cycle than typically observed. This suggested ClpC activity may increase the expression of EB-associated genes. Consistent with this, targeted RT-qPCR analyses demonstrated a significant increase in several EB-associated gene transcripts earlier in development. These effects were not observed with overexpression of the ATPase mutant of ClpC, providing strong evidence that the activity of ClpC drives secondary differentiation. By analyzing the global transcriptional response to ClpC overexpression using RNA sequencing, we observed a shift to earlier expression of canonical late developmental cycle genes and other EB-associated genes. Finally, we directly linked overexpression of ClpC with earlier production of infectious chlamydiae. Conversely, disrupting normal ClpC function with an ATPase mutant caused a delay in developmental cycle progression. Overall, these findings provide the first mechanistic insight for initiation of secondary differentiation in *Chlamydia*.

**IMPORTANCE:**

*Chlamydia* species are obligate intracellular bacteria that require a host cell in which to complete their unique developmental cycle. *Chlamydia* differentiates between an infectious but non-replicating form, the elementary body, and a non-infectious but replicating form, the reticulate body. The signals that drive differentiation events are not characterized. We hypothesize that proteases are essential for mediating differentiation by allowing remodeling of the proteome as the organism transitions from one functional form to another. We previously reported that the Caseinolytic protease (Clp) system is essential for chlamydial growth. Here, we reveal a surprising function for ClpC, an unfoldase, in driving production of infectious chlamydiae during the chlamydial developmental cycle.

## INTRODUCTION

From microbes to mammals, how organisms undergo developmental transitions is a fascinating area of study. This is equally true for the developmentally regulated, obligate intracellular bacterial pathogen, *Chlamydia trachomatis. Chlamydia* species share a developmental cycle in which they transition between an infectious but non-dividing form, the elementary body (EB), and a non-infectious but replicating form, the reticulate body (RB). The intermediate body (IB) is a transitional form as the RB converts into an EB. *Chlamydia* initiates infection when an EB attaches to and uses a type 3 secretion system (T3SS) to secrete effectors into a target cell to induce uptake into a host-derived vesicle, termed the inclusion, that is rapidly dissociated from the endolysosomal pathway (1, 2). *Chlamydia* completes its developmental cycle within this inclusion . Once internalized, the EB immediately begins primary differentiation to the RB (3). *Chlamydia* RBs replicate by an asymmetric polarized budding mechanism, which utilizes peptidoglycan only at the division septum (4–6). After multiple rounds of RB replication, during which the organisms continue to secrete effectors into the host cell and the inclusion membrane, secondary differentiation from the RB to EB is initiated in an asynchronous manner, with IBs and EBs accumulating within the inclusion. EBs are subsequently released from the host cell to infect other nearby cells. The signals that trigger differentiation events between these forms are not characterized.

Temporal transcription profiles of the chlamydial developmental cycle have been investigated since the early 2000s to identify critical factors associated with developmental transitions. Various approaches, including targeted RT-PCR, microarrays (7, 8), and, more recently, RNA sequencing analysis (9, 10), have shown that chlamydial development can be broadly classified into three temporal gene expression patterns coincident with EB-to-RB (i.e., early), RB growth and division (i.e., mid), and RB-to-EB (i.e., late) phases. For *C. trachomatis* serovar L2, a commonly used lab strain, a standard developmental cycle in cell culture takes ~48 hours, after which widespread cell lysis occurs to release EBs for a new round of infection. Early-cycle genes are transcribed between 0 and 8 hours post infection (hpi), where gene function revolves around establishing the inclusion and completing primary differentiation. Mid-cycle genes peak in transcription at ~12–16 hpi and function to mature the inclusion, to acquire nutrients from the host, and to replicate the bacteria. A recent study has suggested that mid-cycle genes may in fact be transcribed much earlier in the developmental cycle (11). Late-cycle genes are transcribed after ~16 hpi and function primarily in secondary differentiation to prepare the EB for the next round of infection. Examples of late genes include those encoding histone-like proteins (12), glycogen metabolism enzymes (13), select T3SS components and effectors (14), and outer membrane proteins (15), among others (12, 13, 15–19). Despite these temporal profiling experiments, it is still not known what triggers these changes in gene expression, as expression patterns alone have not been enough to explain differences in protein abundance.

Multiple proteomic studies of the EB and RB have been performed with the goal to determine key factors that may be associated with these developmental forms (20–24). However, these studies detected relatively few developmental-form-specific proteins. Rather, they observed that the abundance of proteins involved in different processes between these morphologically distinct forms differed. For example, proteins involved in translation, cell maintenance (secretion, redox status, cell division, and chaperones), and nucleic acid metabolism have a higher abundance in RBs than EBs, while proteins involved in cell envelope and energy metabolism are enriched in EBs vs RBs (24). Given the differences in the EB and RB proteomes, we hypothesize that protein turnover is essential for differentiation and the chlamydial developmental cycle.

Recently, our labs investigated the chlamydial ClpX and ClpC orthologs (25–28). ClpX and ClpC are AAA+ unfoldases that use ATP to unfold a target substrate protein into the ClpP proteolytic barrel for degradation. ClpX and ClpC are highly homologous among chlamydial species, indicating that their function is likely highly conserved. Interestingly, overexpressing wild-type ClpX had no phenotypic effect on chlamydial growth, whereas overexpressing an ATPase mutant disrupted the production of EBs (27). Unlike for ClpX, we demonstrated that overexpression of wild-type or catalytically inactive isoforms of ClpC resulted in severe growth defects albeit with different phenotypes (25). More specifically, we observed that overexpression of wild-type ClpC blocked inclusion growth, whereas overexpression of a catalytically inactive mutant of ClpC (ClpCmut) blocked EB production while having limited effects on inclusion morphology. These data suggest that altering the amount of functional ClpCP complex impacts developmental cycle progression. Based on this, we hypothesize that ClpC has a direct role in regulating secondary differentiation.

To test our hypothesis, we compared the effects of overexpressing wild-type or mutant isoforms of ClpC on chlamydial developmental cycle progression. Our data reveal that overexpressing wild-type ClpC prematurely accelerates developmental cycle progression, whereas overexpressing the catalytically inactive mutant had the opposite effect of delaying developmental cycle progression. Three key metrics associated with late stages of development were used to evaluate this: glycogen production, late-cycle transcripts, and production of infectious organisms. These data are the first to provide a mechanism for how *Chlamydia* triggers secondary differentiation and indicate a critical function for ClpC activity in this process.

## RESULTS

### Overexpression of wild-type ClpC_6xH results in accumulation of glycogen within the inclusion lumen

**Fig 1:**
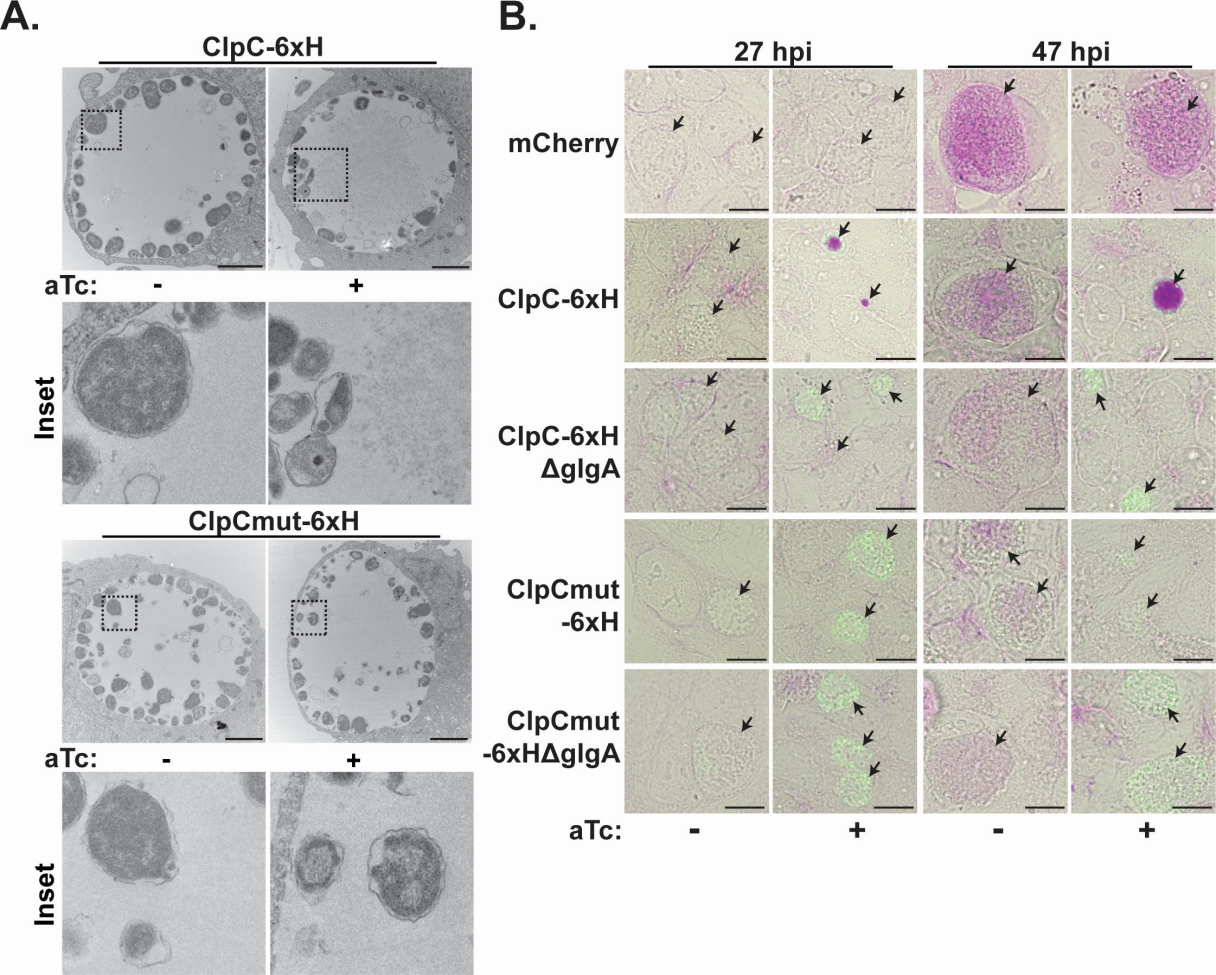
Glycogen accumulates within the inclusion earlier in development during overexpression of ClpC_6xH. (**A**) TEM of *C. trachomatis* serovar L2 overexpressing ClpC_6xH or ClpCmut_6xH. HeLa cells were infected with the indicated strains, and at 10 hpi, expression of the construct was induced or not with aTc. Samples were processed for TEM at 24 hpi. Inset images are digitally zoomed. (**B**) Periodic-acid Schiff staining of HeLa cells infected with wild-type or *glgA* knockout *Chlamydia* expressing mCherry, ClpC_6xH, or ClpCmut_6xH. Expression of the constructs was induced or not at 10 hpi then fixed with MeOH at 27 hpi (*N* = 3) or 47 hpi (*N* = 2). Representative images were taken on a Nikon Eclipse Ti-E microscope using a 100× oil objective. Green = 6xH, magenta = Schiff reagent. Arrows in (**B**) point to inclusions. Scale bar: (**A**) 2 µm and (**B**) 10 µm.

Based on our prior work (25), we sought to evaluate how overexpression of wild-type ClpC disrupted chlamydial growth and development. Here, we used overexpression vectors encoding spectinomycin resistance and validated that these strains exhibited the same phenotypes as our previously described strains encoding β-lactamase (Fig. S1) (25). As an initial strategy to examine phenotypic effects associated with ClpC overexpression, we prepared samples for ultrastructural analysis using transmission electron microscopy (TEM). HeLa cells were infected with the wild-type or mutant ClpC overexpression strain, and expression of ClpC_6xH or ClpCmut_6xH was induced or not at 10 hpi using 20 nM anhydrotetracycline (aTc). The infected cells were fixed and processed for TEM at 24 hpi. As expected based on our prior data, overall bacterial numbers were reduced in the wild-type ClpC_6xH overexpression condition as compared to the control (i.e., uninduced) or ClpCmut_6xH conditions (Fig. 1A; Fig. S2). Unexpectedly, however, overexpression of ClpC_6xH resulted in an aggregation of electron-dense material within the inclusion lumen that was not present in the inclusion lumen of the control conditions. Based on work from others in the field (13), we hypothesized that this aggregated material was glycogen. Glycogen synthesis genes, such as *glgA*, are transcribed as late-cycle genes, and glycogen accumulates within the inclusion lumen at late timepoints in the developmental cycle, where a majority of glycogen is made from the bacterium and a minor amount is transported into the inclusion lumen from the host cell (13, 29–31).

Next, we used a periodic acid-Schiff (PAS) protocol to determine if the aggregate observed during ClpC_6xH overexpression was a polysaccharide. Briefly, periodic acid will oxidize the glycol group of carbohydrates to make dialdehydes, which can bind Schiff’s reagent resulting in a magenta-colored complex (13, 30). HeLa cells were infected with the ClpC_6xH, ClpCmut_6xH, or mCherry overexpression strains, induced or not at 10 hpi, fixed using methanol at 27 or 47 hpi, and stained using the PAS protocol (Fig. 1B). Consistent with the aggregate being glycogen, PAS-positive staining was detected in the ClpC_6xH overexpression conditions from 27 and 47 hpi. We did not observe PAS-positive staining during ClpCmut_6xH overexpression at either timepoint, whereas PAS-positive staining was only detected in the uninduced controls at 47 hpi, a time when glycogen is typically detected within inclusions (32). These results indicate that the detection of PAS-positive staining, particularly at the earlier timepoint, is associated with ClpC activity.

To test whether the aggregated polysaccharide material was glycogen, we generated *Ctr* serovar L2 *glgA* (CtrL2*glgA::bla*; involved in glycogen synthesis) and *glgP* (CtrL2*glgP::bla*; involved in glycogen breakdown) TargeTron knockout strains containing β-lactamase (*bla*) insertion cassettes to disrupt these genes (Fig. S3). We then created CtrL2*glgA::bla* and CtrL2*glgP::bla* strains carrying the *aadA* containing ClpC_6xH or ClpCmut_6xH overexpression plasmids (Fig. 1B; Fig. S4). As a control, we also created knockout strains carrying a plasmid encoding inducible mCherry. Loss of *glgA* resulted in minimal, likely from the host cell, or no PAS-positive staining within the inclusion lumen of any of the strains regardless of expression of a ClpC isoform or mCherry, while loss of *glgP* showed a general increase in glycogen staining for all strains and conditions at 47 hpi, likely due to loss of glycogen breakdown. As in a wild-type background, PAS-positive staining was maintained during ClpC_6xH overexpression at 27 hpi in the *glgP* knockout background. These data indicate that wild-type ClpC overexpression results in aggregation of glycogen by a GlgA-dependent mechanism.

### Transcripts for the glycogen synthase gene, *glgA*, are increased earlier in the developmental cycle during ClpC_6xH overexpression

**Fig 2:**
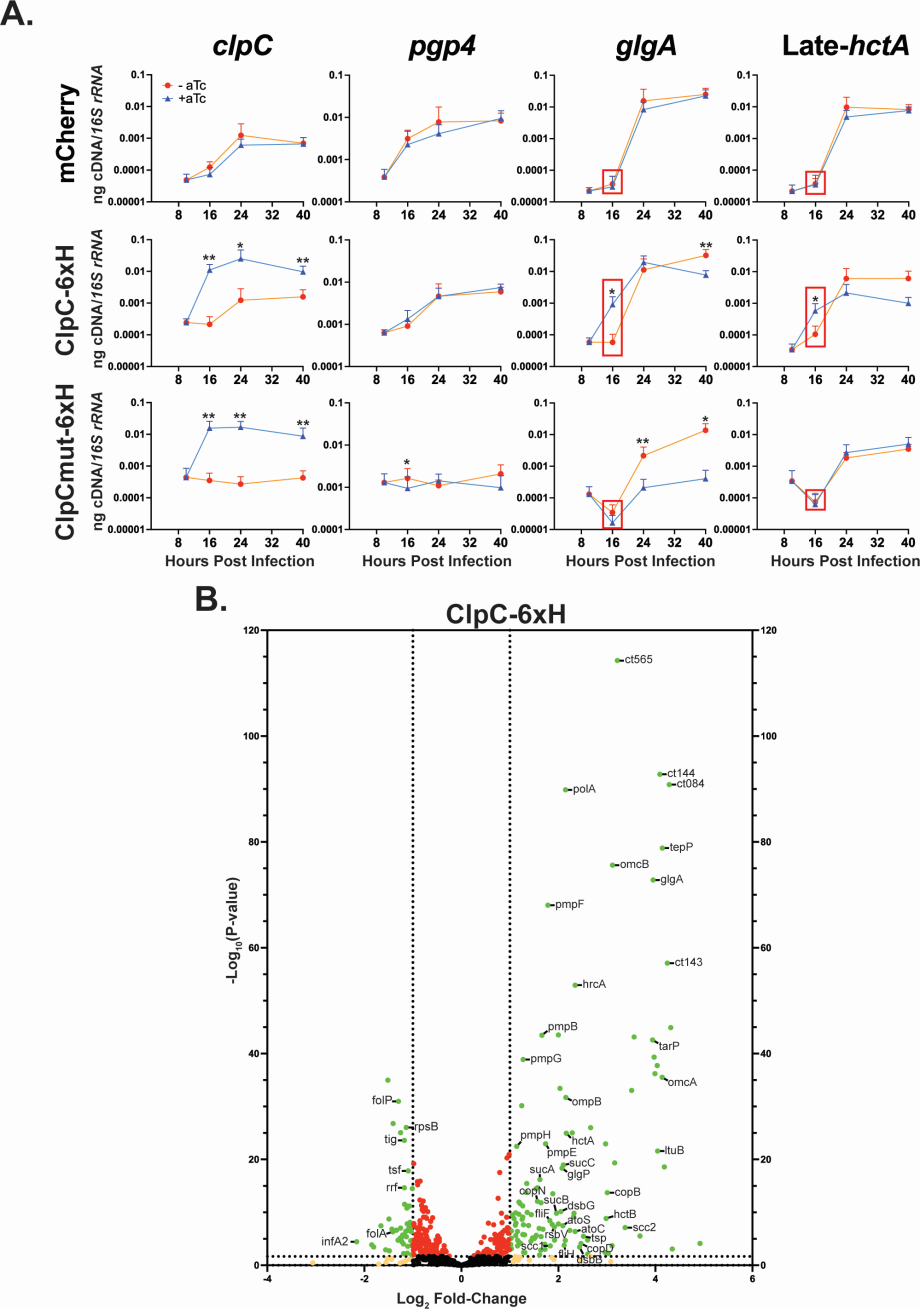
Overexpression of ClpC_6xH globally increases late gene transcripts. (**A**) RT-qPCR measuring *clpC*, the plasmid-specific gene *pgp4*, *glgA*, and a late gene*-hctA* from *C. trachomatis* L2 overexpressing or not mCherry, ClpC_6xH, or ClpCmut_6xH. HeLa cells were infected, and at 10 hpi, constructs were induced or not with aTc. RNA was collected at 10, 16, 24, and 40 hpi then processed for RT-qPCR. Transcript quantities from cDNA were normalized to *16S rRNA* values. Notably, *glgA* and *hctA* were only increased when ClpC_6xH is overexpressed. Boxes highlight the 16 hpi timepoint at which *glgA* and *hctA* transcripts are typically low, as observed for the mCherry or ClpCmut expressing conditions or all uninduced controls. Statistical significance analysis was performed via a parametric ratio Student’s *t*-test (*P < 0.05; **P < 0.01). (**B**) Volcano plot of RNA-sequencing results from HeLa cells infected with *C. trachomatis* L2 expressing ClpC_6xH as compared to its matched uninduced control. Construct expression was induced or not at 10 hpi, and RNA was collected at 18 hpi and processed for RNA-sequencing. All data are the average of three independent experiments. The vertical dashed lines indicate a fold change of at least two, whereas the horizontal dashed line indicates a false discovery rate *P* value of 0.05. See also Table 1.

To assess why glycogen was accumulating during overexpression of wild-type ClpC_6xH, we quantified transcripts for the glycogen synthesis gene *glgA*, its plasmid-encoded transcriptional regulator *pgp4* (33), as well as a canonical late gene, *hctA* (one of the first EB-specific genes expressed in *Chlamydia* [34]) as a control for late gene expression (Fig. 2A). Cells were infected with the same three transformant strains used for PAS staining, expression of the construct was induced or not at 10 hpi, and nucleic acids were harvested at 10, 16, 24, and 40 hpi. Overexpression of mCherry had no significant impact on any of the transcripts assessed, nor did it affect overall *16S rRNA*, genomic DNA (gDNA), or plasmid DNA (pDNA) levels (Fig. 2A; Fig. S5).

Overexpression of wild-type ClpC_6xH was linked to a reduction in overall *16S rRNA* and gDNA levels while having no significant impact on overall plasmid copy number, consistent with previous immunofluorescence analysis (IFA) and inclusion forming unit (IFU) findings ([25]; Fig. S5). In uninduced control conditions, transcripts for *glgA* increased at 24 hpi, consistent with its late developmental cycle expression. However, we measured a greater than 1-log increase in *glgA* transcripts at 16 hpi (a mid-developmental cycle timepoint) when overexpressing ClpC_6xH compared to the uninduced control. There were no differences in transcript levels for the *glgA* plasmid-encoded transcriptional regulator, Pgp4 (Fig. 2A). To determine if other Pgp4-regulated genes were upregulated, we examined an additional gene: *pls1* (33). We found *pls1*, another late gene, also had ~1 log increased transcript levels at 16 hpi during ClpC_6xH overexpression that did not reach statistical significance (*P* = 0.079). We also examined genes involved in glycogen breakdown, *glgP* and *glgX*, and observed no changes in transcript levels for these genes (Fig. S6A). Unexpectedly, we measured elevated transcripts of *hctA* at 16 hpi (Fig. 2A), suggesting that other late genes may also be upregulated earlier in the developmental cycle. Overall, these data support that accumulation of glycogen at 27 hpi during ClpC_6xH overexpression is linked to earlier expression of *glgA*.

Overexpression of ClpCmut_6xH resulted in no changes in *16S rRNA*, gDNA, or pDNA levels compared to uninduced controls (Fig. S5). However, *glgA* and *pls1* transcripts were significantly reduced at 24 and 40 hpi, whereas no meaningful differences were observed for *pgp4* or *hctA* transcripts (Fig. 2A; Fig. S6A). We also measured decreased transcript levels for other late genes, including *hctB*, *tarP*, and *lcrH1/scc2* (Fig. S6B). These data explain the absence of PAS-positive staining when overexpressing ClpCmut_6xH (Fig. 1B).

### Overexpression of ClpC_6xH causes a global upregulation of late genes

The RT-qPCR data showed elevated *hctA*, *glgA*, and *pls1* transcripts at 16 hpi, suggesting that transcription related to secondary differentiation may be altered by ClpC activity. To determine if overexpression of ClpC is sufficient to trigger transcription of late genes more generally, we performed RNA-sequencing (RNAseq) during ClpC_6xH overexpression (Fig. 2B). Cells were infected with the ClpC_6xH overexpression strain, induced or not at 10 hpi, and RNA was collected at 18 hpi and processed for RNAseq (quality analysis of reads provided in Table S1). The 18 hpi timepoint was chosen for this experiment with the rationale that late gene transcription is activated after 16 hpi and is nearing peak activity by 24 hpi. Therefore, if we detected increased late gene transcripts during ClpC_6xH overexpression above and beyond the uninduced control, then this would further strengthen ClpC’s role in activating late gene transcription.

Using a stringent twofold cut-off to compare the induced to the uninduced conditions, statistical analysis of the results identified 119 genes that displayed increased transcripts and 50 genes that displayed decreased transcripts under conditions of ClpC_6xH overexpression (Table S2). Among the most downregulated genes were those associated with RB growth, including translation-linked genes, such as *infA*, *tsf*, and multiple ribosomal genes, and metabolism-linked genes, such as *folAPX* and *atpA*. To investigate the link between ClpC activity and EB production, we next evaluated the upregulated transcriptional changes in six categories linked to secondary differentiation (Table 1; full data in Tables S2 to S7): “canonical” late genes, glycogen-associated genes, Pgp4-regulated genes, *pmp* (polymorphic outer membrane protein; autotransporter protein) genes, genes involved in regulation, and “other” late genes. Canonical late genes are those that are well established in the literature as being associated with EBs and having characterized transcriptional patterns associated with secondary differentiation. Glycogen-associated genes were included given the observed phenotype of increased glycogen staining earlier during the developmental cycle when overexpressing ClpC_6xH (for the glycogen pathway gene list, see Table S3). The expression of *glgA* is regulated by Pgp4, which also regulates five additional genes (*ct049* [*pls1*], *ct084*, and the *ct142-ct144* operon), which serve as non-essential virulence factors during late development (33). These genes were included as the Pgp4 regulon. The *pmp* genes were included since there is a compositional change in the bacterial membranes between the RB and EB (for a full list of membrane-associated genes, see Table S4). We also included gene regulation-associated genes since this class may impact the expression of other genes. “Other” late genes, mostly hypotheticals, were determined based on prior RNAseq studies from our lab comparing transcript levels at 24 hpi to those at 10 hpi (35).

**TABLE 1:** Partial list of genes significantly increased during ClpC_6xH overexpression^*a*^

Category	CT no.	CTL no.	Name	Function	Fold change	References
Canonical late genes	CT444	CTL0703	*omcA*	Small cysteine-rich outer membrane protein	17.63	(15, 36, 37)
	CT080	CTL0336	*ltuB*	Late transcription unit B protein	16.52	(17)
	CT798	CTL0167	*glgA*	Glycogen synthase	15.48	(7)
	CT456	CTL0716	*tarP*	Translocated actin-recruiting phosphoprotein	15.38	(38)
	CT576	CTL0839	*scc2 (lcrH_1*)	Type III secretion chaperone (low calcium response protein H)	10.38	(7, 39)
	CT443	CTL0702	*omcB*	Large cysteine-rich outer membrane protein	8.67	(7, 17)
	CT578	CTL0841	*copB*	Needle tip; translocator	8.05	(7, 39)
	CT046	CTL0302	*hct2 (hctB*)	Histone H1-like protein HC2	7.91	(7, 12)
	CT441	CTL0700	*tsp*	Carboxy-terminal processing protease	5.72	(40)
	CT579	CTL0842	*copD*	Needle tip; translocator	5.53	(7, 39)
	CT176	CTL0428	*dsbB*	Probable disulfide formation protein	5.41	(7, 18)
	CT743	CTL0112	*hctA*	Histone H1-like protein HC1	4.48	(7)
	CT177	CTL0429	*dsbA*	Disulfide bond chaperone	4.14	(7, 18)
Glycogen-associated genes	CT798	CTL0167	*glgA*	Glycogen synthase	15.48	
	CT087	CTL0342	*malQ*	4-alpha-glucanotransferase	4.42	
	CT248	CTL0500	*glgP*	Alpha-1,4 glucan phosphorylase	4.22	
	CT489	CTL0750	*glgC*	Glucose-1-phosphate adenylyltransferase	2.28	
Pgp4 regulated	CT084	CTL0339		Phosphatidylcholine-hydrolyzing phospholipase D (PLD) protein	19.51	
	CT143	CTL0398		Uncharacterized protein	18.99	
	CT144	CTL0399		Putative membrane protein	17.06	
	CT142	CTL0397		Uncharacterized protein	15.91	
	CT049	CTL0305	pls1	Uncharacterized protein	15.69	
	CT798	CTL0167	glgA	Glycogen synthase	15.48	
*pmp* Genes	CT869	CTL0248	pmpE	Polymorphic outer membrane protein	3.33	
	CT871	CTL0250	pmpG	Polymorphic outer membrane protein	2.42	
	CT872	CTL0251	pmpH	Polymorphic outer membrane protein	2.21	
	CT870	CTL0249	pmpF	Polymorphic outer membrane protein	3.44	
	CT413	CTL0670	pmpB	Polymorphic outer membrane protein	3.16	
	CT412	CTL0669	pmpA	Polymorphic outer membrane protein	−2.15	
Regulatory genes	CT468	CTL0728	*atoC*	Two-component system response regulator	5.08	
	CT675	CTL0044	*mcsB*	Protein-arginine kinase	4.00	
	CT467	CTL0727	*atoS*	Two component regulator, histidine kinase	3.77	
	CT676	CTL0045	*mcsA*	McsB activator protein	3.66	
	CT424	CTL0683	*rsbV1*	Anti-sigma factor antagonist	3.04	
	CT630	CTL0894	*chxR*	Atypical response regulator protein	1.92	
Other RNA-seq-based late genes	CT084	CTL0339		Phosphatidylcholine-hydrolyzing phospholipase D protein	19.51	
	CT073	CTL0329		Exported protein	18.18	
	CT144	CTL0399		Putative membrane protein	17.06	
	CT382.1	CTL0638		Uncharacterized protein	16.41	
	CT049	CTL0305		Uncharacterized protein	15.69	
	CT132	CTL0387		Uncharacterized protein	12.83	
	CT051	CTL0307		Uncharacterized protein	11.80	
	CT702	CTL0071		Uncharacterized protein	11.40	
	CT546	CTL0808		Uncharacterized protein	8.94	
	CT392	CTL0648		Uncharacterized protein	8.61	

^*a*^ Both naming schemes for *Ctr* serovar D (CT) and L2 (CTL) are presented. “Name” is the annotated gene name under the accession file (other common names are in parentheses). Genes in each category are either complete (i.e., representing all genes in that category that were increased in transcript levels) or the top 10-highest fold changed genes in their respective categories. Genes listed in Pgp4-Regulated are those characterized by the Caldwell lab in 2013 (33). We identified these genes as among the top 13 statistically significant upregulated genes when inducing expression of ClpC_6xH. CT142–CT144 are in an operon. “Other RNA-seq based late genes” were characterized based on at least a twofold increase in transcript levels between 10 and 24 hpi during a *C. trachomatis* L2 wild-type infection (see Ouellette et al. [35]). See Supplementary Tables for more details and gene hits within the categories listed as well as Fig. 2 for experimental details.

Consistent with our targeted RT-qPCR analysis, transcripts of the canonical late genes were globally increased during ClpC_6xH overexpression as indicated in Table 1. These include gene products involved in condensation of the chromosome (*hctA* [4.48×] and *hctB* [7.91×] [12]), in periplasmic remodeling via protease activity (*tsp* [40]), in outer membrane crosslinking (*dsbB* [5.41×], *dsbA* [4.14×], *omcA* [17.63×], and *omcB* [8.67] [15, 18]), in T3SS (*scc2/lcrH_1* [10.38×] , *copB* [8.05×], and *copD* [5.53×] [39]) or effectors (*tarp* [15.38×] [38, 41]) for priming the bacteria for their next infection, and in unknown pathways (*ltuB* [16.53×] [17]). Unsurprisingly, we observed upregulation of *glgA* (15.48×), and transcripts for other glycogen-associated genes, *malQ* and *glgP*, were upregulated approximately fourfold (for all glycogen pathway genes, see Table S3). The Pgp4 regulon was upregulated as expected based on the changes in *glgA* transcripts. Most of the *pmp* genes were increased during ClpC_6xH overexpression, and some of these have been characterized as EB associated (15).

*Chlamydia* encodes a single two-component signaling system that has been linked to secondary differentiation via regulation of the alternative sigma factor, σ^54^. The response regulator encoded by *atoC*, the protein of which activates σ^54^-controlled gene expression (42), and its histidine kinase encoded by *atoS* were both increased, thus providing a mechanistic link between ClpC_6xH overexpression and the putative σ^54^ regulon (42, 43). Intriguingly, transcripts for *mcsA* (3.66-fold) and *mcsB* (fourfold), the products of which are associated with marking proteins for degradation by ClpC (44–47), were also increased. Finally, based on our prior RNAseq experiments, we prepared a list of late genes based on at least a twofold increase from 10 to 24 hpi (Table S5). From this list, 65 predicted late genes, mostly hypotheticals, were significantly upregulated by more than twofold. Of the 119 significantly increased genes, 46 were secretion associated. These included homologs to: T3SS apparatus (11), chaperones involved in T3SS (5), T3S effectors that are inserted into the inclusion membrane (Incs) (3), known effectors that are secreted into either the host or the inclusion lumen (14), putative exported proteins (6), or uncharacterized proteins suggested to be secreted by experiments in heterologous systems (7) (Tables S6 and S7) (48). Some of our hits included several Incs, such as *incV* (2.92×), known to stabilize inclusion interactions with other organelles such as the endoplasmic reticulum (49). Interestingly, one of the uncharacterized proteins (CT849) increased more than any other gene, 30.3-fold, suggesting the factor(s) regulating its expression is likely a substrate of ClpC.

Overall, these data suggest that ClpC_6xH overexpression may trigger secondary differentiation earlier in development, observed as a global increase in late gene expression and abnormally early production of glycogen in the inclusion lumen.

### Increased ClpC_6xH levels result in increased late gene transcripts when bacterial replication is blocked

**Fig 3:**
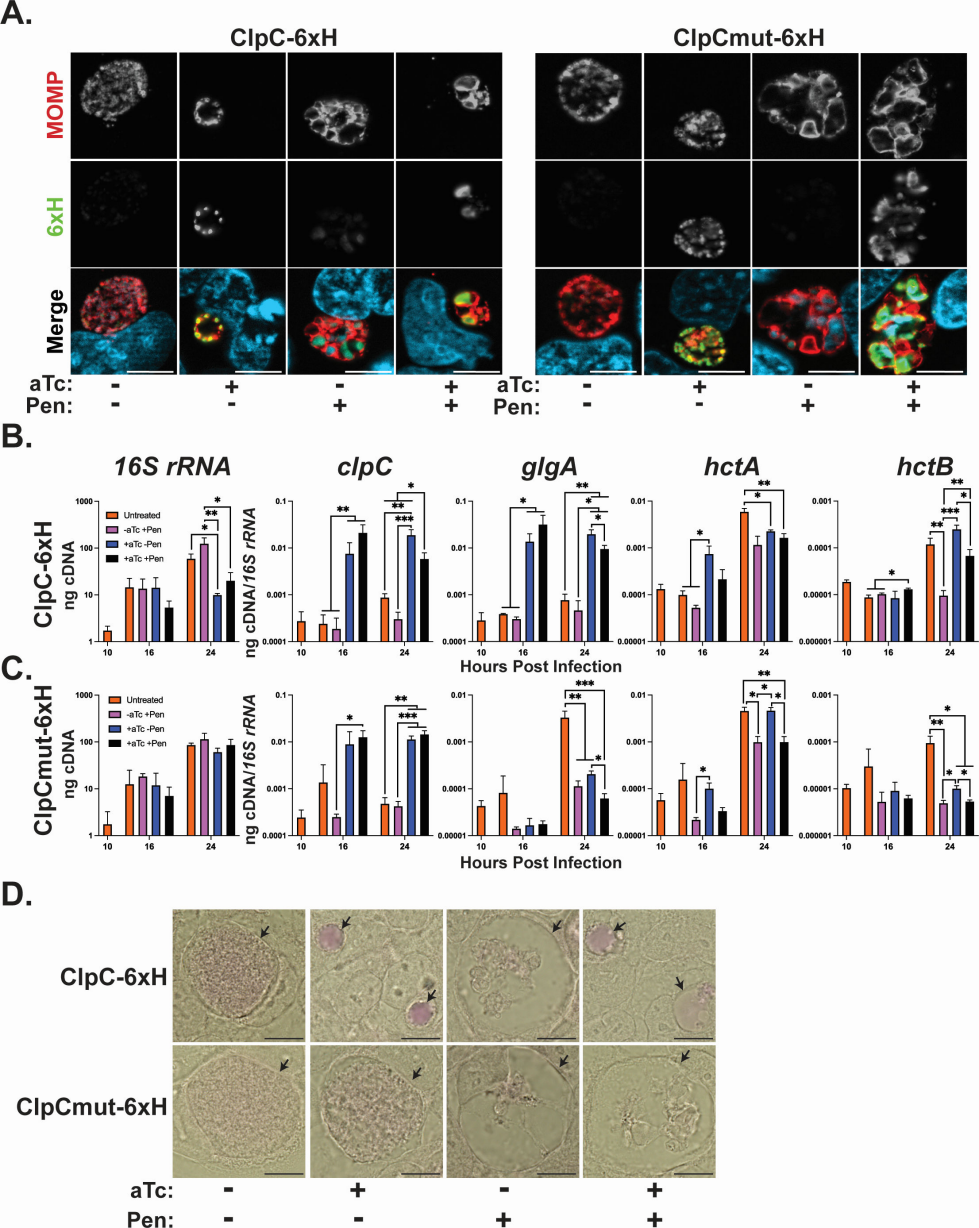
Overexpression of ClpC_6xH increases late gene transcripts when the developmental cycle is blocked. HeLa cells were infected with *C. trachomatis* overexpressing ClpC_6xH or ClpCmut_6xH. At 10 hpi, constructs were induced or not with aTc and treated or not with penicillin (Pen). (**A**) Visualization of inclusion morphology by indirect immunofluorescence analysis. Infected cells were fixed with MeOH at 24 hpi and stained for MOMP (red), 6xH (green), and DNA (light blue). Representative images were taken on a Zeiss Apotome at ×100 magnification with ×5.5 digital zoom. Scale bar = 5 µm. (**B and C**) RT-qPCR of *16S rRNA* and *clpC*, and late genes *glgA*, *hctA*, and *hctB* during ClpC_6xH (**B**) or ClpCmut_6xH (**C**) overexpression under the indicated conditions at 10, 16, and 24 hpi. Data are shown as bars where: untreated = orange, +Pen = magenta, +aTc = blue, and +aTc + Pen = black. Transcripts were normalized to *16S rRNA*. For ClpC_6xH (**B**), notably, the late genes *glgA* and *hctB* were similarly expressed between the +aTc – Pen and +aTc + Pen at 24 hpi compared to Pen treatment alone (i.e., –aTc + Pen). Data were log-transformed, and statistical analysis was performed using a repeated measures two-way ANOVA with the Geisser-Greenhouse correction and Tukey’s multiple comparison test with individual variances computed for each comparison (*P < 0.05; **P < 0.01; ***P < 0.001). (**D**) Visualization of glycogen by periodic-acid Schiff staining. At 48 hpi, infected cells were fixed with MeOH and stained using PAS (*N* = 3). Representative images were taken on a Nikon Eclipse Ti-E microscope using a 100× oil objective. Magenta = Schiff reagent. Arrows point to inclusions. Scale bar: 10 µm.

We reasoned that, if ClpC activity is able to activate the transcription of genes involved in secondary differentiation, overexpressing ClpC_6xH under conditions when the developmental cycle is blocked should similarly activate these late genes. To test this, we performed RT-qPCR as described previously, but with the additional condition of penicillin (Pen) treatment. Of note, if peptidoglycan synthesis is blocked by penicillin, then RB division is halted, EB-associated gene expression is not activated, and no EBs are produced (50, 51). Morphologically, penicillin-treated *Chlamydia* is grossly enlarged and often has non-uniform distribution of its major outer membrane protein (MOMP). After infecting a monolayer of HeLa cells, ClpC_6xH expression was induced or not with aTc and treated or not with Pen at 10 hpi, where Pen treatment alone would serve as our negative control for blocked late gene transcription. As with our prior experiments, we used spectinomycin-resistant strains that are susceptible to Pen. IFA controls at 24 hpi validated the expected bacterial morphology under the varying treatment conditions (Fig. 3A). RNA was collected at 10, 16, and 24 hpi and processed for RT-qPCR (Fig. 3B). We measured that *16S rRNA* transcripts were unaffected in bacteria treated with Pen, as previously reported (50), while these transcripts were decreased by approximately one log in the aTc and aTc + Pen groups. Similarly, the addition of aTc resulted in an approximately 1.5 log increase in *clpC* transcripts for both aTc alone and aTc + Pen compared to the uninduced and +Pen alone conditions.

We next determined if ClpC_6xH expression was sufficient to activate late genes during Pen treatment. We measured transcripts of the known late genes *glgA*, *hctA*, and *hctB* under various treatment conditions. Consistent with our prior experiments, overexpression of ClpC_6xH without Pen treatment (blue bars) resulted in an increase in *glgA* and *hctA* transcripts earlier in development at 16 hpi. In the uninduced control without Pen treatment (orange bars), these transcripts were increased by 24 hpi, following their normal developmental expression pattern. Conversely, for the uninduced control treated with Pen (magenta bars), we observed that transcripts for *glgA* and *hctB* remained near or below a basal level (i.e., 10 hpi value), consistent with prior reports (50). Interestingly, *hctA* transcripts increased in all conditions by 24 hpi, suggesting that Pen treatment does not affect transcript levels of this particular late gene. When ClpC_6xH expression was induced in the presence of Pen (black bars), we measured a significant increase in *glgA* transcripts at 16 hpi similar to ClpC_6xH expression alone and a significant increase in *hctB* transcripts at 24 hpi similar to uninduced and untreated conditions; in other words, ClpC_6xH overexpression resulted in late gene transcription during Pen treatment. Importantly, this increase in late gene expression during Pen treatment was not associated with EB production (Fig. S7). We also tested ClpCmut_6xH under the same conditions and observed that inducing ClpCmut_6xH expression in the presence of Pen did not significantly change transcript levels of late genes as compared to Pen treatment alone (Fig. 3B).

We next tested whether the increase in *glgA* transcripts during aTc + Pen treatment was sufficient to generate glycogen (Fig. 3D). Similar to our previous PAS-staining results (Fig. 1B), both the uninduced and the aTc-induced samples, in the absence of Pen, produced PAS-positive staining at 48 hpi. As expected, treatment with Pen only was not sufficient to obtain PAS-positive inclusions at 48 hpi since late gene, and more specifically *glgA*, expression was blocked. However, by inducing ClpC_6xH during Pen treatment, we observed PAS-positive inclusions at 48 hpi, indicating the accumulation of glycogen in these conditions. As expected, the expression of ClpCmut_6xH did not result in PAS-positive inclusions under any of the conditions tested. These data suggest that, by increasing ClpC activity during Pen treatment, these bacteria can activate, at least partially, late gene transcription with the associated phenotype of glycogen production.

### Overexpression of ClpC_6xH results in earlier production of infectious chlamydiae

**Fig 4:**
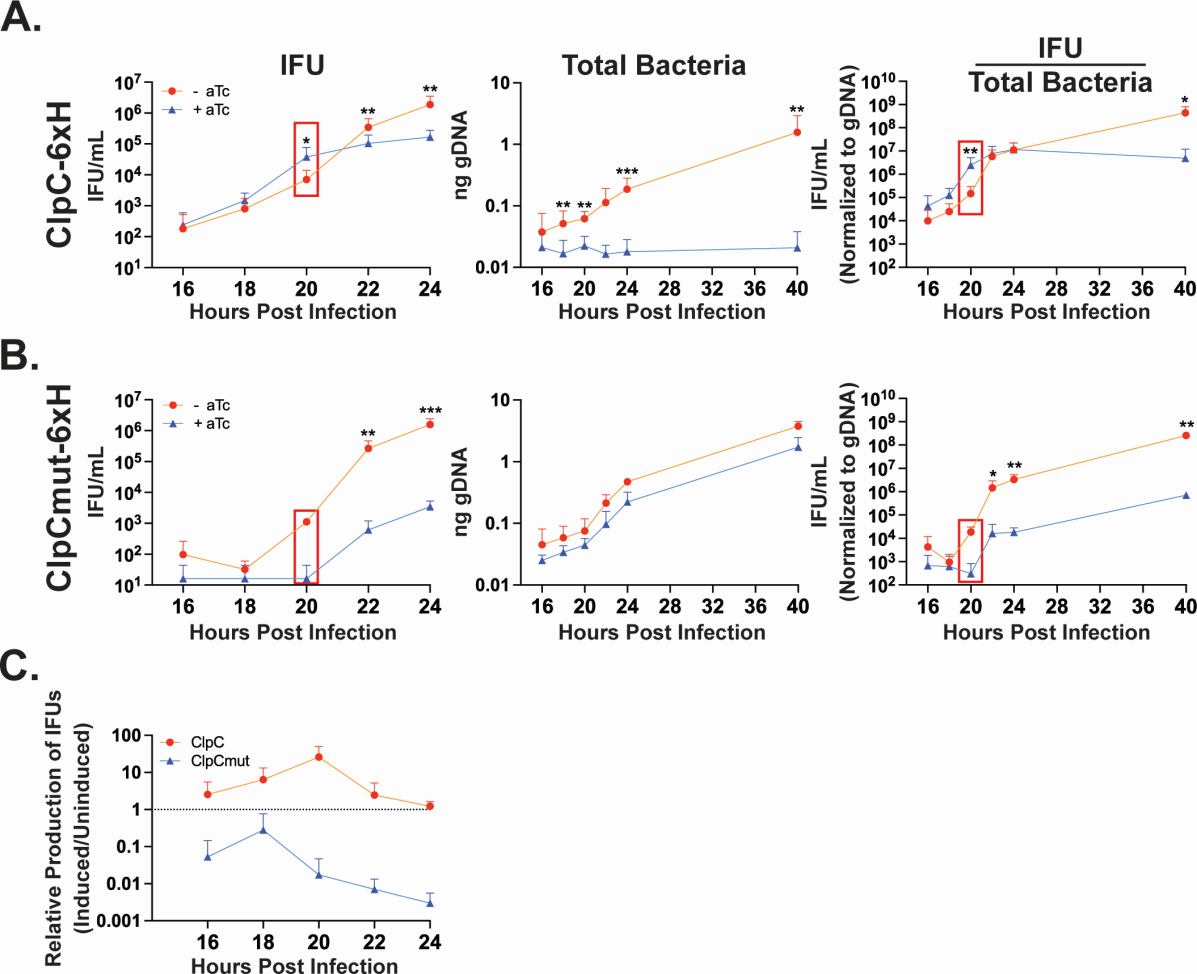
Overexpression of ClpC_6xH results in an earlier increase in IFU production. One-step growth curves of HeLa cells infected with *C. trachomatis* L2 overexpressing or not (**A**) ClpC_6xH or (**B**) ClpCmut_6xH. At 10 hpi, constructs were induced or not with aTc. Infected cell lysates were collected between 16–24 and 40 hpi for either secondary infection to determine the amount of viable infectious organisms (IFU/mL), gDNA, for total bacteria (RBs + EBs), or IFU/mL normalized to gDNA to give the relative amounts of IFUs within the total population (IFU/[RB + EB]). Notice the boxes at 20 hpi where induced ClpC_6xH surpasses the uninduced value for IFU/mL, while the gDNA value remains significantly lower for the induced compared to uninduced sample. (**C**) Percent IFUs in induced cultures as compared to uninduced cultures was calculated using the IFU/mL normalized to gDNA from (**A**) or (**B**), thus comparing the percentage of IFUs between the induced to uninduced conditions. This value indicates that ClpC activity directly correlates to the percent of infectious bacteria produced. Statistical significance analysis was performed via a parametric, ratio Student’s *t*-test (*P < 0.05; **P < 0.01; ***P < 0.001).

The sum of our data thus far indicates that overexpression of ClpC_6xH prematurely activates global late gene transcription, and this is associated with at least one phenotypic marker of the late developmental cycle—glycogen accumulation. However, the “gold standard” for measuring secondary differentiation is the IFU (a measure of infectious chlamydiae) assay. We predicted that, if ClpC_6xH overexpression were triggering premature secondary differentiation, then we should be able to measure an earlier increase in IFUs. Complicating this analysis, however, is that fewer RBs undergoing secondary differentiation would result in overall lower IFU yields. Nevertheless, we investigated this by infecting HeLa cells with the wild-type or mutant ClpC_6xH inducible expression strains. Expression of the constructs was induced or not at 10 hpi, and IFU and gDNA samples were collected every 2 hours between 16 and 24 hpi as well as at 40 hpi to assess overall IFU yield near the end of a standard developmental cycle (Fig. 4). Although gDNA levels were significantly decreased throughout development when inducing ClpC_6xH, we measured a steady increase in IFUs, indicating bacteria were viable and progressing through secondary differentiation into infectious chlamydiae (Fig. 4A). In contrast, the uninduced control produced IFUs at a steady rate that increased logarithmically, along with total gDNA, throughout the developmental cycle, as expected. The IFU data normalized to gDNA (i.e., the fraction of total bacteria that are infectious chlamydiae) indicated that ClpC_6xH overexpression triggers earlier production of IFUs (at least within 6 hours of induction), which is completed around 24 hpi as these values plateau. During these conditions, IFUs were approximately 1-log higher at 20 hpi compared to the uninduced control conditions. The observation that glycogen accumulation did not occur during overexpression of ClpCmut_6xH led us to perform the same analysis for IFUs for this strain (Fig. 4B). Here, there were no significant differences in total bacteria, as represented by gDNA levels, when overexpressing ClpCmut_6xH as compared to the uninduced control. However, IFUs, normalized or not to gDNA levels, remained extremely low, showing more than a 3-log decrease by the end of a standard developmental cycle (Fig. 4B). Similar results were observed when knocking down *clpC* transcripts by CRISPRi (Fig. S8). To compare the two overexpression strains more directly, we graphed the relative levels of IFUs using the ratio of IFUs produced as a function of total bacterial numbers during induced conditions compared to uninduced conditions (Fig. 4C). Any value above 1 indicates more IFUs produced under inducing conditions. This analysis revealed that ClpC_6xH overexpression resulted in increased IFU production from 16 to 22 hpi. Conversely, overexpression of ClpCmut_6xH resulted in decreased IFU production throughout the time course. Overall, these analyses indicate that the activity of ClpC is essential for secondary differentiation and linked to IFU production.

## DISCUSSION

In the current study, we examined the effects of overexpressing wild-type or mutant ClpC isoforms on chlamydial developmental cycle progression. Our data show that increased levels of wild-type ClpC resulted in accelerated developmental cycle progression. We made this conclusion based on three principal findings: earlier accumulation of glycogen within the inclusion lumen, earlier increases in late gene transcript levels, and earlier production of infectious organisms (IFUs). Conversely, overexpressing a mutant isoform of ClpC that lacks catalytic activity blocked developmental cycle progression, resulting in no glycogen accumulation, decreased late gene transcription, and significantly impaired IFU production. We attempted to quantify developmental forms by TEM under conditions when ClpC_6xH was overexpressed or not. However, given the smaller sizes of the inclusions during ClpC_6xH overexpression and the limitations of TEM vis-à-vis sample processing and image acquisition, we were unable to perform a quantitative analysis of developmental forms. Nonetheless, our data from three separate metrics support a function for ClpC activity in driving developmental cycle progression in *Chlamydia*.

**Fig 5:**
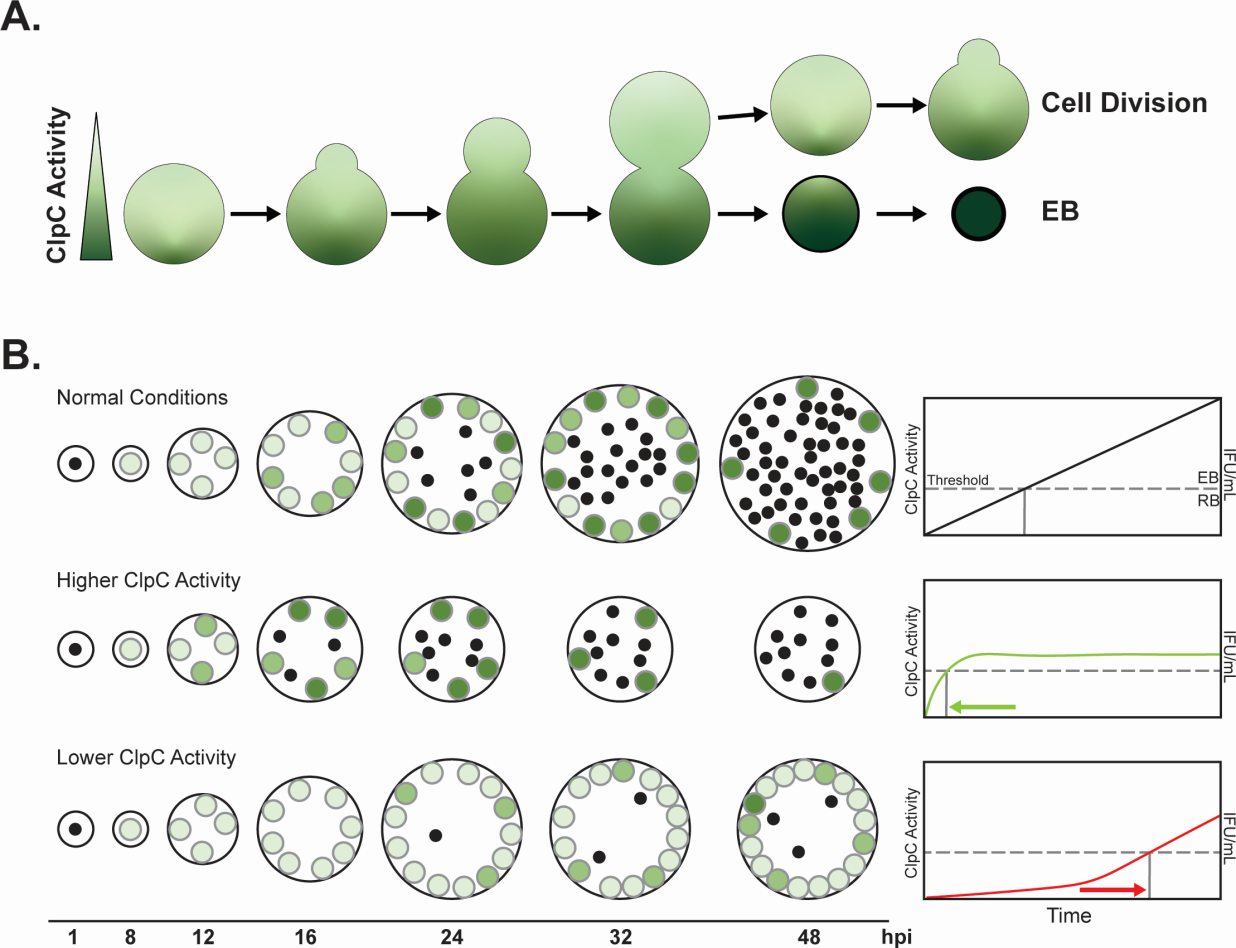
Model of the impact of ClpC activity on secondary differentiation. (**A**) As *Chlamydia* divides by an asymmetric polarized division process, we speculate that the levels of ClpC are unequally distributed between mother and daughter cells. This leads to different fates for each cell after septation, consistent with the asynchronous nature of chlamydial secondary differentiation. (**B**) Developmental representation of ClpC activity (left). An earlier increase in ClpC activity results in premature conversion of RBs to EBs, resulting in smaller inclusion size and fewer overall bacteria. A decrease in ClpC activity results in a similar inclusion size as in normal conditions but with a decrease in EBs. Effect of ClpC activity on secondary differentiation (right). Dashed line represents the threshold of ClpC activity required for secondary differentiation to occur (i.e., RB to EB). Lines represent ClpC activity (left y-axis) with IFU/mL output (right y-axis). The x-axis indicates time.

Secondary differentiation is an essential process for all chlamydial species, yet the mechanisms that govern this process are poorly understood. It is possible, for example, that this process is regulated by some combination of intra- or intercellular signaling between the host environment, within which *Chlamydia* grows, and the bacterium itself. In fact, with its complex growth requirements, it is reasonable to speculate that *Chlamydia* integrates multiple signal inputs before committing to this major event. We, therefore, propose a threshold that must be overcome for any signal to trigger secondary differentiation. This signal threshold would explain the asynchronous nature of secondary differentiation since local conditions of any given RB will determine if and when it commits to an EB. Here, as ClpC activity (either the amount of ClpC or its complex with ClpP) increases past a threshold, an unknown substrate(s) is degraded below a critical threshold, thus allowing activation of late genes and secondary differentiation. Alternatively, if ClpC should function more as a chaperone, then its increased levels may activate a factor(s) that similarly drives secondary differentiation. The unfolding and/or activation of a target substrate by ClpC are not mutually exclusive, and both may be necessary to drive the bacterium toward secondary differentiation. In contrast, when ClpC activity is decreased—either by destabilizing the complex with the ClpCmut_6xH isoform, which is unable to unfold substrates, or by decreasing the levels of ClpC by CRISPRi-mediated knockdown—this unknown substrate(s) is not degraded and/or activated thus maintaining an RB-like state. *Chlamydia* may undergo several replication cycles while degrading/activating the critical substrate(s) before its levels are sufficiently depleted and/or activated by ClpCP to initiate EB production. We predict that this would occur stochastically within any individual RB, allowing some RBs to continue to divide while others commit to secondary differentiation (Fig. 5). This would be consistent with the asynchronous nature of secondary differentiation in *Chlamydia* and a recent modeling experiment (34).

Previous studies have attempted to identify triggers of differentiation in *Chlamydia*, but none have had sufficient experimental evidence to support the hypothesis. For example, glutamine has been suggested as a trigger for EB to RB conversion based on morphological changes to the EB when exposed to this amino acid in axenic medium (52). Changes in the phosphoproteome between EBs and RBs have also been noted, and this could be linked to differential gene expression (53). In *Bacillus subtilis*, dephosphorylation of the anti-anti-sigma factor RsbV by RsbU allows RsbV to bind the anti-sigma factor RsbW, thus releasing its cognate sigma factor to activate genes. *Chlamydia* has orthologs to RsbV (RsbV1 and RsbV2) and RsbW, which, unusually, regulate its major sigma factor, σ^66^ (54). This in turn may depend on phosphorylation of RsbV1 and link to the availability of ATP. Release of σ^66^ allows upregulation of primarily RB-specific genes, including those related to metabolic processes (for review, see reference 53). To this end, work from the Hefty lab suggested TCA intermediates may serve as ligands for RsbU (55), and Rosario and Tan provided evidence that phosphoenolpyruvate, made by enolase through glycolysis and converted to pyruvate by pyruvate kinase, can inhibit the phosphatase activity of RsbU *in vitro* (56). These studies link the Rsb pathway to ATP availability via glycolysis and the TCA cycle. Of note, ATP acquisition differs between the two developmental forms. RBs produce ATP via a sodium gradient, and EBs generate ATP from stored glucose and from the host (13, 57). Therefore, the generation of an ion gradient may drive differentiation depending on energy availability or lack thereof. However, no effect on altering late gene expression was demonstrated from manipulation of these systems, suggesting the Rsb pathway alone is not sufficient to trigger differentiation.

Previously, we reported on the Clp protease system and its function in chlamydial growth and development (25–28). In contrast to what we observed with ClpC, overexpression of the wild-type ClpP orthologs or ClpX, another AAA+ unfoldase, did not impact chlamydial growth or development (25–27). Nonetheless, we recently observed that ClpX, which recognizes SsrA-tagged proteins and mediates their degradation by ClpXP complexes, is critical for secondary differentiation (28). The inability of *Chlamydia* to degrade SsrA-tagged proteins resulted in RBs that were unable to differentiate to EBs (28). This was investigated by (i) using a ClpX mutant unable to target SsrA tags for degradation and (ii) altering the SsrA tag such that it is inefficiently recognized by ClpX. In these strains, late gene transcription was activated, showing post-translational regulation of secondary differentiation is integral to EB formation. Protein turnover by proteases is one such post-translational mechanism that may help regulate developmental transitions in *Chlamydia*.

One intriguing possibility is that ClpC inactivates a critical transcription factor such as EUO to promote late gene expression. However, to date, we have been unable to demonstrate an interaction between ClpC and EUO (unpublished observation). EUO has been shown *in vitro* to bind some promoters associated with EB production (58). Though overexpression of EUO has previously been studied, knockdown of EUO *in vivo* has not. We would note, however, that, in *C. pneumoniae*, late gene transcripts were increased during conditions in which EUO levels were also increased (50); thus, it is not clear whether EUO is sufficient to act as a negative regulator of late genes since this function should be conserved across chlamydial species. In *Chlamydia*, the expression of *glgA* is regulated through transcriptional activation by the plasmid-based protein, Pgp4 (33). However, we did not measure a significant change in plasmids per bacteria or *pgp4* transcript levels when overexpressing ClpC (Fig. S5). Therefore, it was unexpected that all genes regulated by Pgp4 were increased in transcript levels (Table 1). Pgp4 regulates six different genes (*glgA*, *ct049*, *ct084*, and the *ct142–ct144* operon), which serve as non-essential virulence factors during late development (33). These all fell within the top 13 most significantly upregulated hits, suggesting ClpC may function to activate Pgp4 (Table 1). This was supported by the effects of overexpressing ClpCmut_6xH, where *glgA* transcripts were significantly lower, and *pgp4* transcripts similarly did not change between uninduced and induced conditions. This implies that the ATPase activity of ClpC is required for Pgp4 activation. We are currently investigating this. Nonetheless, since the plasmid itself is not required for chlamydial growth and development and is not conserved across chlamydial species (31, 59), Pgp4 cannot be a critical mediator of secondary differentiation.

Clearly, the next step is to identify the substrates of this system and to test whether ClpC acts as a chaperone toward a given substrate or unfolds it for degradation. We are currently developing the necessary protocols to do this in an obligate intracellular bacterium. Of further interest, *Chlamydia* has retained the arginine kinase McsB along with its cognate activator McsA. In other bacteria, McsB has been shown to mark proteins for degradation by ClpC (44–47). Therefore, *Chlamydia* may detect arginine phosphorylated proteins for degradation by ClpCP, thus initiating secondary differentiation. To determine the consequences of phosphorylating these proteins, we will next investigate not just the broad substrates of ClpC but also the McsB-directed phosphoproteome that is targeted by ClpC. In sum, we describe the first experimental evidence for a factor, in this case, ClpC activity, that can directly influence developmental cycle progression in *Chlamydia*.

## MATERIALS AND METHODS

### Strains and cell culture

Human epithelial HeLa cells were used for all studies generating data (overexpression, RNA, gDNA, etc.). HeLa cells were routinely passaged at 37°C, 5% CO_2_ in Dulbecco’s modified Eagle’s medium (DMEM; Gibco/Thermo Fisher) with 10% fetal bovine serum (FBS; Sigma, St. Louis, MO) at 37°C, 5% CO_2_ and were verified to be Mycoplasma free by a LookOut Mycoplasma PCR Detection kit (Sigma). *C. trachomatis* serovar L2 EBs (25,667R; kind gift of Dr. Ian Clarke, University of Southampton) that lacked endogenous pL2 plasmid were prepared and used for transformation (31). All chemicals and antibiotics were obtained from Sigma unless otherwise noted.

### Plasmid construction

A full list of primers used in this study is provided in Table S8 within the supplemental material. Constructs made for chlamydial transformation were created using the HiFi Cloning protocol (New England Biolabs [NEB]; Ipswich, MA). NEBuilder assembly tool was used to design primers for PCR amplification of target genes with a poly-histidine (6xH) tag to the gene of interest being inserted on the 3′ end of the overlap into the vector. *C. trachomatis* L2 (Bu 434) genomic DNA was used as template for PCR amplification, and products were confirmed for correct size by agarose gel electrophoresis. Overexpression plasmids were generated by first taking PCR amplified products and inserting them into the backbone of pBOMBL_Pen_::L2 or pBOMBL_Spc_::L2 (60) cut with EagI and KpnI (Fast digest enzymes; Thermo) to remove the mCherry gene and treated with alkaline phosphatase (FastAP; Thermo). The HiFi reactions were transformed into DH10b *Escherichia coli* (NEB), and the isolated plasmid was verified by restriction enzyme digest and Sanger sequencing by Azenta Life Sciences (Burlington, MA). The sequence-verified plasmids were transformed into *C. trachomatis* (see below).

### Chlamydial transformation

For transformation, 10^6^ *C. trachomatis* serovar L2 EBs (25,667R) naturally lacking the endogenous L2 plasmid (-pL2) were incubated at room temperature with 2 µg of plasmid in a volume of 50 µL Tris-CaCl_2_ (pH 7.5) for 30 minutes following the method developed by Mueller et al. (61). The transformation mix was added to a 2 mL overlay of room temperature DMEM containing 10 µg/mL gentamicin and 10% FBS, per well. The plate was centrifuged at 400 × *g* for 15 minutes at room temperature and placed in an incubator at 37°C. At 8 hpi, medium was removed and replaced with DMEM containing 1 µg/mL cycloheximide, 10 µg/mL gentamicin (Gibco/Thermo), 500 µg/mL spectinomycin (pBOMBL_Spc_) or 2 units/mL penicillin (pBOMBL_Pen_), and 10% FBS. Infected cells were passaged every 48 hours by scraping cells from the plate in a 2 mL microcentrifuge tube and centrifuging at 17,000 × *g*. The pellet was resuspended in 500 µL sucrose-phosphate (2SP) (31) and centrifuged again at 400 × *g* to remove host cell debris. Two hundred fifty milliliters of inoculum was added dropwise to 2 mL of DMEM containing 1 µg/mL cycloheximide, 10 µg/mL gentamicin, 500 µg/mL spectinomycin or 2 units/mL penicillin, and 10% FBS on a new monolayer of HeLa cells, plated 1 day prior at 1 × 10^6^ cells per well. If a population of spectinomycin-resistant and GFP-positive bacteria was established, then EBs were harvested as described above and frozen at −80°C prior to titration. Plasmids were isolated from the strains using the DNeasy kit (Qiagen), re-transformed into *E. coli*, and re-isolated to verify insert by digest and sequencing.

### Preparation of samples for transmission electron microscopy

HeLa cells were plated in a six-well plate the day before infection (1 × 10^6^ HeLa cells per well) at two wells per strain. The following day, they were infected at an MOI = 1 with either pBOMBL_Spc_-*clpC_6xH*::L2 or pBOMBL_Spc_-*clpCmut_6xH*::L2 containing strains and induced or not with 20 nM aTc at 10 hpi. These strains were verified to have the same phenotypes as previously published *bla*-encoding pBOMBL strains (Fig. S1, [25]). At 24 hpi, medium was aspirated, and cells were washed with Dulbecco’s phosphate-buffered saline (dPBS) then trypsinized to detach them from the plate. The infected cells were collected, centrifuged at 400 × *g* for 5 minutes, washed with ice-cold Hanks’ balanced salt solution (HBSS) a total of three times, and finally resuspended in 500 µL of electron microscopy fixative (2% glutaraldehyde, 2% paraformaldehyde, and 0.1 M phosphate buffer) provided by the University of Nebraska Medical Center electron microscopy core facility.

### TargeTron mutagenesis of *glgA* and *glgP*

The TargeTron mutagenesis of *glgA* (CTL_0167) and *glgP* (CTL_0500) was completed using the parental strain *C. trachomatis* L2 (434 Bu). The methods of Johnson and Fisher (62) were followed to achieve the site-specific inactivation of *glgA* or *glgP*, and the *C. trachomatis* L2 parental strain was used as a control for confirmation experiments. The modification of the pACT vector was accomplished with *glgA*-targeting oligonucleotides IBS1/2_*glgA*, EBS1_*glgA*, and EBS2_*glgA*, while the modification of the pACT vector was accomplished with *glgP*-targeting oligonucleotides, IBS1/2_*glgP*, EBS1_*glgP*, and EBS2_*glgP*.

These digested TargeTron vectors were ligated with the targeting sequences and transformed into *dam^–^*/*dcm*^–^ *E. coli* (NEB). Plasmid was isolated and confirmed by sequencing.

For the transformation of *C. trachomatis* with the TargeTron vectors, a mixture of 6 µg of the purified plasmid DNA and density-gradient purified L2/434/Bu EBs was added to sterile buffer for transformation (10  mM Tris, pH 7.5, and 50 mM CaCl_2_). After incubating this mixture for 30 minutes at room temperature, it was added to each well of monolayers of L929 cells on a six-well plate that was prewashed with HBSS. The plate was centrifuged (545 × *g*, 1 h, 20°C), then the inoculum was removed, and Dulbecco’s modified Eagle medium plus 10% FBS (DMEM-10; Corning, Inc., Corning, NY, USA) was added. After incubating the plates (37°C in 5% CO_2_ for 5 h), the media was replaced by DMEM-10 containing 1 mM cycloheximide and 10 IU/mL of penicillin G. Cultures were passaged as above until penicillin-resistant EBs were obtained from the monolayers.

The preliminary screening of the *glgA*-interrupted strains was performed by PCR by using oligonucleotide primers pET26b-*glgA* forward and pET26b-*glgA* reverse, while screening of the *glgP*-interrupted strains was performed using oligonucleotide primers pET26b-*glgP* forward and pET26b-*glgP* reverse. The genome sequencing for both the mutant strains and the parent strain was completed following the method described by DeBoer et al. (63).

### Generation of Δ*glgA* or Δ*glgP* strains carrying pBOMBL_spc_ plasmids

*C. trachomatis* L2 *glgA*::GII(*bla*) and *C. trachomatis* L2 *glgP*::GII(*bla*) strains were generated by TargeTron insertion into the lab strain *C. trachomatis* L2 (see above). To transform these knockout strains with an overexpression plasmid, a confluent monolayer of HeLa cells in a six-well plate, plated 1 day prior at 1 × 10^6^ cells per well, was infected with combinations of either pBOMBL_Spc_-*mCherry*::L2, pBOMBL_Spc_-*clpC_6xH*::L2, or pBOMBL_Spc_-*clpCmut_6xH*::L2 chlamydial transformants and *Ctr*L2Δ*glgA::bla* or *Ctr*L2Δ*glgP::bla* at an MOI = 2 for each strain in DMEM with 10 µg/mL gentamicin and 10% FBS. This method relied on the exchange of plasmids between strains within an infected host cell. After sufficient time had passed for plasmid exchange (16 hpi), media was aspirated and replaced with DMEM containing 1 µg/mL cycloheximide, 10 µg/mL gentamicin, 250 µg/mL spectinomycin, 0.5 U/mL penicillin, and 10% FBS. Newly generated strains were passaged 10 times and confirmed to have the appropriate *glgA* and *glgP* knockouts by PCR as well as the pBOMBL plasmid (see Table S8 for primers).

### Periodic acid-Schiff staining for glycogen

Strains carrying pBOMBL_Spc_-*mCherry*::L2, pBOMBL_Spc_-*clpC_6xH*::L2, or pBOMBL_Spc_-*clpCmut_6xH*::L2 with or without Δ*glgA* or Δ*glgP* were used to infect a confluent monolayer of HeLa cells (seeded the previous day at 2 × 10^5^ cells per well) in a 24-well plate with a glass coverslip in each well. At 10 hpi, infected wells were induced or not with 20 nM aTc for the *Ctr*L2 strains, or 2 nM aTc for the *Ctr*L2 *glgA* or *glgP* knockout strains. At time of collection, media was aspirated, and cells were washed with dPBS or HBSS and then fixed with MeOH for 10 minutes. After fixing, coverslips were washed three times with dPBS + 0.025% Sodium Azide. The following glycogen stain was adapted from Schaart et al. (64). Briefly, 250 µL of 1% periodic acid was added per well and incubated for 5 minutes. Periodic acid was removed, and coverslips were placed in tap water for 1 minute and then rinsed with MilliQ pure water. Water was removed and replaced with 250 µL of Schiff’s reagent (Electron Microscopy Services, Hatfield, PA) per well then incubated at room temperature for 15 minutes. Schiff’s reagent was removed, and coverslips were washed with a series of MilliQ and tap water rinses until sections turned a darker pink. Tap water was removed then coverslips were washed three times with MilliQ water. Glycogen staining was not detected with less than 40× magnification for 40 hpi uninduced samples. Coverslips were subsequently labeled with goat anti-MOMP primary antibody (Meridian Bioscience, Memphis, TN) and rabbit anti-6xH (Abcam; Cambridge, MA). Donkey anti-goat Alexa Fluor 405-conjugated secondary antibody (Invitrogen/Thermo/Jackson Labs) was used for visualization of the organisms for experiments. A donkey anti-mouse Alexa Fluor 488-conjugated secondary antibody (Invitrogen/Thermo/Jackson Labs) was used for visualization of 6xH expression. Lastly, samples were stained with 4’,6-diamidino-2-phenylindole (DAPI; Sigma) to visualize the host and bacterial DNA. Of note, MOMP and DAPI were imaged on the same channel since oxidation of cells bleaches the near- and far-red wavelengths. Representative images were taken on a Nikon Eclipse Ti-E microscope using a 100× oil objective and processed with NIS elements software (V5.21; Nikon, Melville, NY).

### Validation of pBOMBL_Spc_ strains via immunofluorescence and inclusion-forming unit analysis

*C. trachomatis* transformed with pBOMBL_Spc_-*mCherry*::L2, pBOMBL_Spc_-*clpC_6xH*::L2, or pBOMBL_Spc_-*clpCmut_6xH*::L2 under the control of an aTc-inducible promoter were used to infect a monolayer of HeLa cells on coverslips utilizing 500 µg/mL spectinomycin as a selection agent. Samples were induced or not with 20 nM aTc at 10 hpi and fixed at 24 hpi with methanol (ClpC_6xH and ClpCmut_6xH) for 10 minutes, or 3.25% formaldehyde and 0.025% glutaraldehyde for 2 minutes followed by MeOH for 10 minutes (mCherry: to maintain fluorescence). Samples prepared for assessment of blocked cell division (Fig. 3; Fig. S7) were additionally treated or not with 10 U Pen/mL at 10 hpi. Fixed cells were incubated with goat anti-MOMP primary antibody (all IFA samples; Meridian Bioscience, Memphis, TN) and rabbit anti-6xH (ClpC_6xH and ClpCmut_6xH; Abcam; Cambridge, MA). Donkey anti-goat Alexa Fluor 594-conjugated (for 6xH strains) or 488-conjugated (for mCherry strain) secondary antibody (Invitrogen/Thermo or Jackson Labs) were used to visualize MOMP (organisms). A donkey anti-rabbit Alexa Fluor 488-conjugated secondary antibody (Invitrogen/Thermo/Jackson Labs) was used to visualize ClpC_6xH and ClpCmut_6xH expression. Lastly, samples were stained with DAPI (Sigma) to visualize host and bacterial DNA. Representative images were taken on an Axio ImagerZ.2 equipped with Apotome.2 using a 100× objective and then 5.5× digitally zoomed and adjusted equally for color and brightness with Zen software (blue edition, V3.3).

To assess the effects of wild-type and ClpC mutant overexpression, the IFU assay was used. HeLa cells were infected in triplicate as described above for IFA samples. In each IFU well, cells were harvested by scraping in 250 µL 2SP and pooling triplicate samples together before freezing at −80°C. Samples were serially diluted in HBSS and used to infect duplicate wells of HeLa cells (seeded the day before at 2 × 10^5^ cells per well). At 24–40 hpi, GFP fluorescent inclusions from 10 fields of view were counted for each well at 20× magnification, giving a total of 20 fields of view per experiment. Displayed values are expressed as a percentage of the uninduced sample to provide an internal control from three independent experiments. A parametric, unpaired Student’s two-tailed *t* test was used to compare the induced samples to the uninduced control.

### Analysis of transcripts from overexpression strains

Two wells of a six-well plate, at a density of 10^6^ cells per well, per condition were infected using *C. trachomatis* transformed with either pBOMBL_Spc_-*mCherry*::L2, pBOMBL_Spc_-*clpC_6xH*::L2, or pBOMBL_Spc_-*clpCmut_6xH*::L2 at an MOI of 1. At 10 hpi samples were induced or not with 20 nM aTc. At each time point, RNA was collected using TRIzol reagent (Invitrogen) according to the manufacturer’s instructions. DNA was removed from RNA samples through rigorous DNase treatment (Turbo DNAfree; Thermo Fisher) before 1 µg was reverse transcribed with Superscript III reverse transcriptase (Thermo Fisher). Equal volumes of generated cDNA were used for qPCR. For genomic or plasmid DNA, cells were trypsinized and collected by centrifugation for 5 minutes at 400 × *g* and resuspended in phosphate-buffered saline then stored at −20°C until further processing with the DNeasy Blood and Tissue kit (Qiagen) according to the manufacturer’s instructions. Equal amounts of gDNA were used for qPCR. Transcripts and genomic DNA were quantified by qPCR in 25 µL reaction mixtures using 2× SYBR green master mix (Thermo Fisher) in a QuantStudio3 thermal cycler using a standard curve generated from purified *C. trachomatis* L2 genomic DNA. Genomic and plasmid DNA were quantified using *clpP1* (*Chlamydia* has two ClpP paralogs, ClpP1 and ClpP2) or *pgp4*, respectively. Statistics were performed using a parametric ratio Student’s *t*-test using three biological replicates with three technical replicates each.

### Nucleic acid extraction and enrichment from *Chlamydia* for RNA-sequencing

RNA was collected at 18 hpi from uninduced and induced cultures as described above from HeLa cells infected with pBOMBL_Spc_-*clpC_6xH*::L2. Host mRNA and rRNA were removed from RNA-sequencing samples using MICROB*Enrich* and MICROB*Express* (Invitrogen/ThermoFisher) kits according to the manufacturer’s instructions. Samples were aliquoted and stored at −80°C until submission to the UNMC Genomics Core. Three biological replicates were collected.

### Library preparation and RNA sequencing

RNA quality was assessed via Fragment Analyzer (Advanced Analytical) and Nanodrop. Final libraries were quantified using Qubit DS DNA HS Assay reagents in Qubit Fluorometer (Life Technologies), and the size of libraries was measured via Fragment Analyzer. RNA-seq libraries were prepared from 400 ng RNA using Illumina Ribo-Zero plus Microbiome (Illumina, Inc. San Diego, CA) following the recommended protocol. Resultant libraries from the individual samples were multiplexed and subjected to 100 bp paired read sequencing using an Illumina Novaseq 6000 in the UNMC Genomics Core facility (see Table S1). The DNA library pool was denatured with 0.2N NaOH. The final loading concentration was 300 pM. The original fastq format reads were trimmed by fqtrim tool (https://ccb.jhu.edu/software/fqtrim) to remove adapters, terminal unknown bases (Ns), and low-quality 3′ regions (Phred score <30). The trimmed fastq files were processed by FastQC (65). *C. trachomatis* 434/Bu bacterial reference genomes and annotation files were downloaded from Ensembl (https://bacteria.ensembl.org/Chlamydia_trachomatis_434_bu_gca_000068585/Info/Index). The trimmed fastq files were mapped to *C. trachomatis* 434/Bu by CLC Genomics Workbench 23 for RNA-seq analyses.

### RNA-seq statistical analyses

Each gene’s read counts were modeled by a separate Generalized Linear Model (GLM), assuming that the read counts follow a negative binomial distribution, and were normalized based on transcripts per kilobase per million. The Wald test was used for statistical analysis of the two-group comparisons. The false discovery rate and Bonferroni adjusted *P* values were provided to adjust for multiple-testing problems. Fold changes are calculated from the GLM, which corrects for differences in library size between samples and effects of confounding factors. The quality of reads mapped can be found in Table S1 and statistically analyzed data in Table S2.

### One step growth curve of chlamydial overexpression or knockdown strains

*C. trachomatis* transformed with pBOMBL_Spc_-*mCherry*::L2, pBOMBL_Spc_-*clpC_6xH*::L2, pBOMBL_Spc_-*clpCmut_6xH*::L2, or the dCas12 knockdown transformant pBOMBL12CRia_Spc_(*clpC*)::L2 under the control of an aTc-inducible promoter was used to infect at an MOI of 1 a monolayer of HeLa cells in two wells of a six-well plate. Samples were induced or not at 10 hpi with 20 nM aTc for the pBOMBL_Spc_ strains and 2 nM for the pBOMBL12CRia_Spc_ strain. One well was harvested for IFUs by collecting scraped cells in 1 mL 2SP buffer then freezing overnight at −80°C. IFU samples were titrated as described above. gDNA samples were collected and processed as described above.

## Data Availability

The raw and processed RNA sequencing reads have been deposited in Gene Expression Omnibus (GEO) with accession number GSE255503.

## SUPPLEMENTAL MATERIAL

Supplemental Figures mbio.02870-24-s0001.pdf Fig. S1-S8.

Legends mbio.02870-24-s0002.docx Supplemental figure legends.

Supplemental Tables mbio.02870-24-s0003.xlsx Tables S1-S8.

ASM does not own the copyrights to Supplemental Material that may be linked to, or accessed through, an article. The authors have granted ASM a non-exclusive, world-wide license to publish the Supplemental Material files. Please contact the corresponding author directly for reuse.
