# Supplementary figures and images for: Overexpressing the ClpC AAA+ unfoldase accelerates developmental cycle progression in Chlamydia trachomatis
Source: mBio. 2024 Nov 22;16(1):e02870-24. doi: 10.1128/mbio.02870-24 (PMC11708050; doi:10.1128/mbio.02870-24)

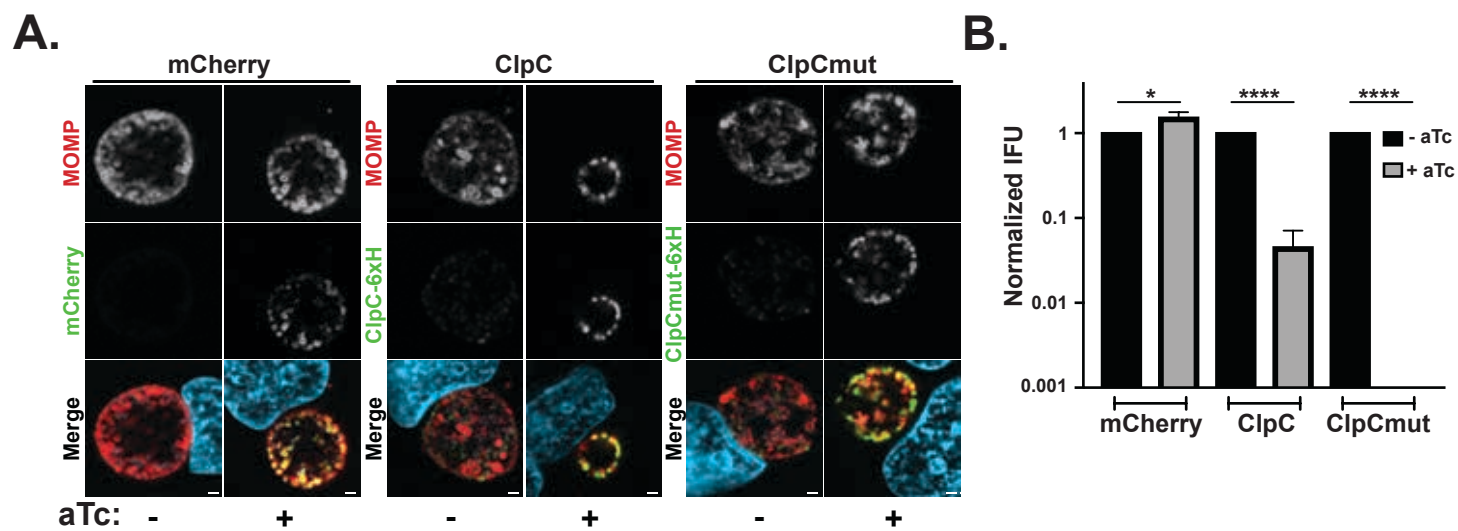

Figure S1

**pBOMBL<sub>Pen</sub>-c/pC-6xH::L2**

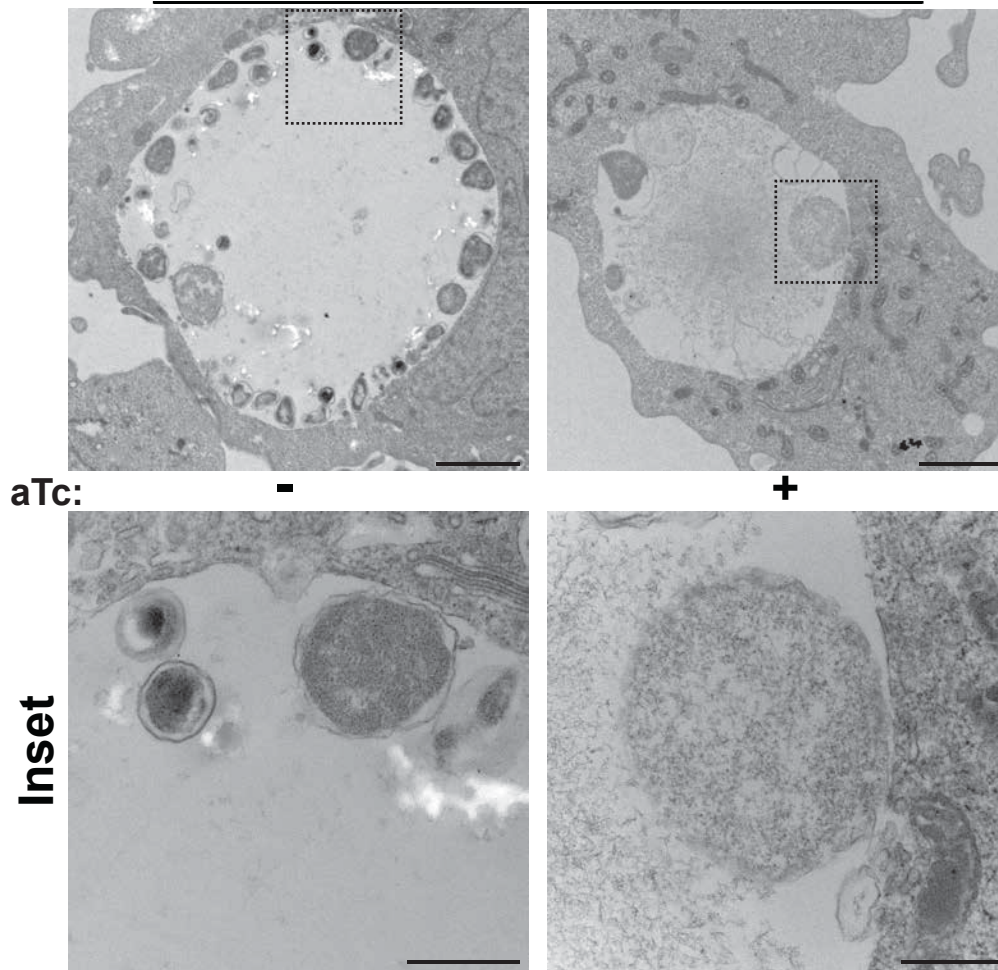

**Figure S2**

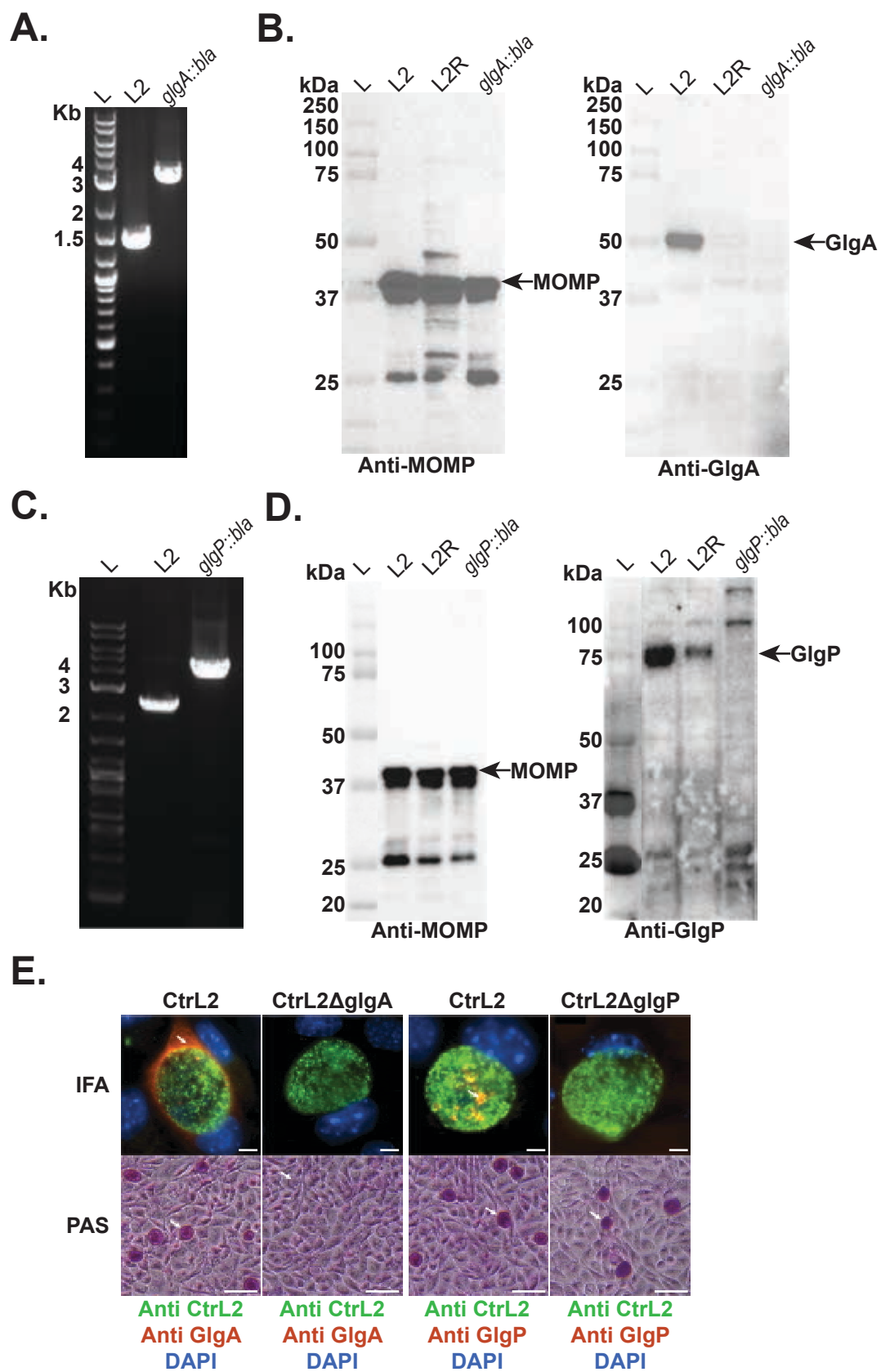

Figure S3

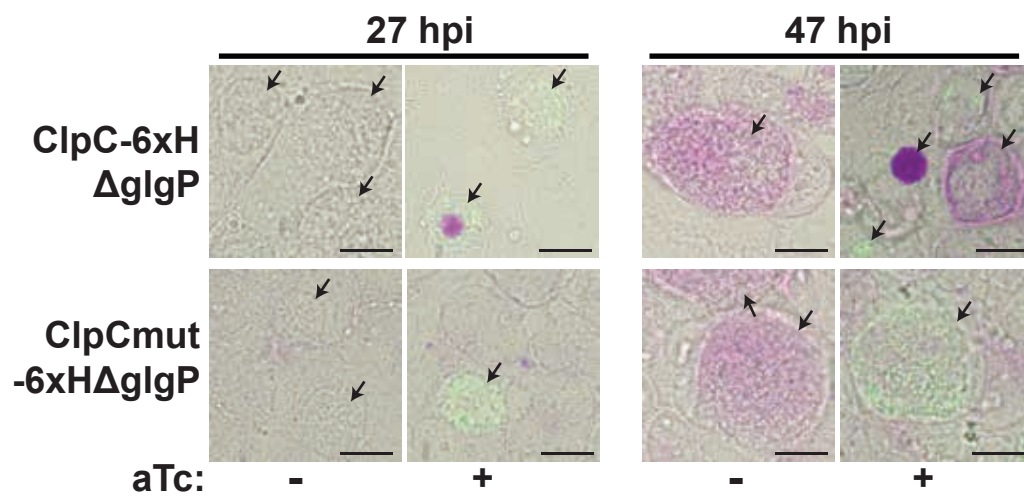

Figure S4

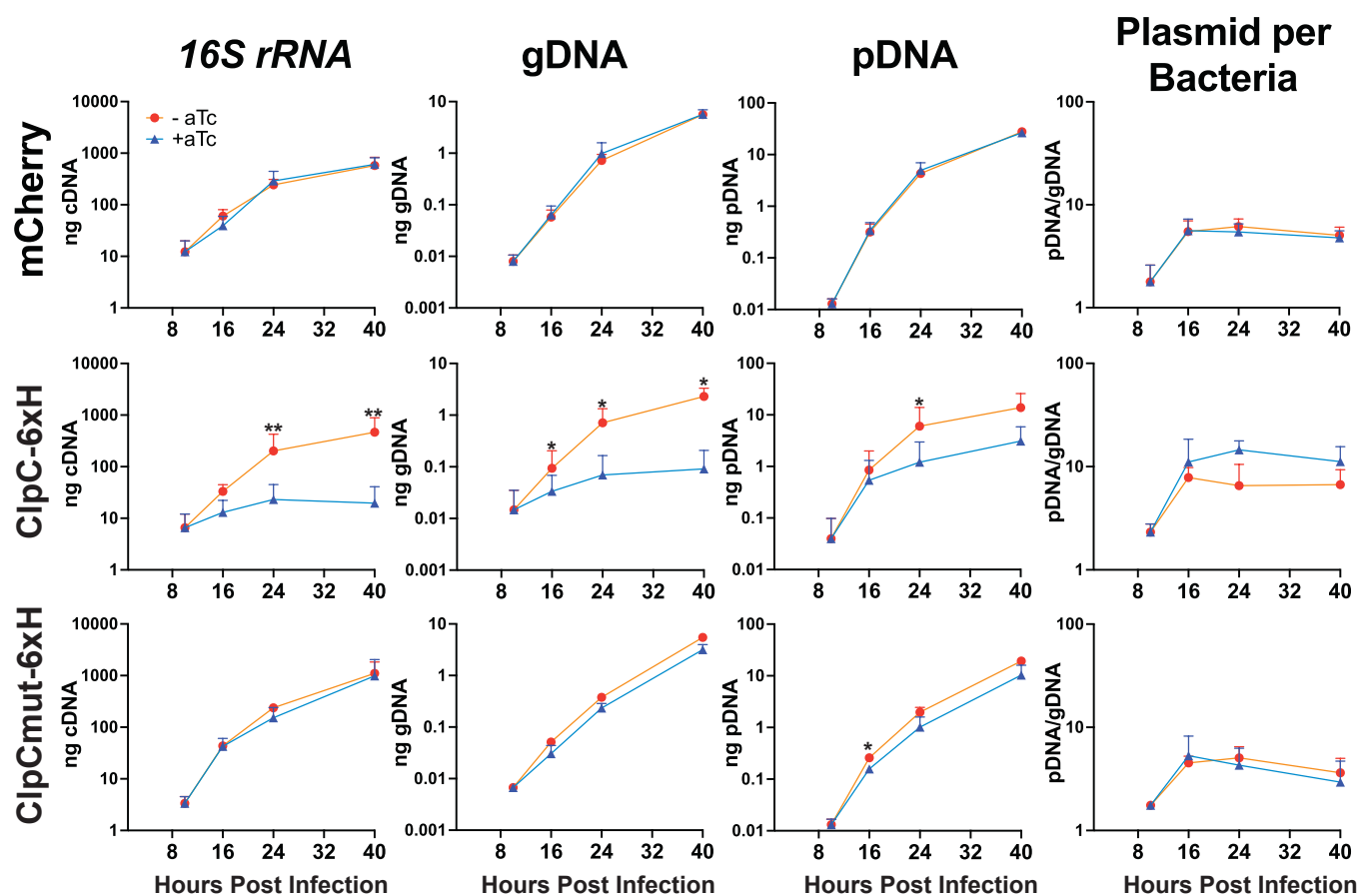

Figure S5

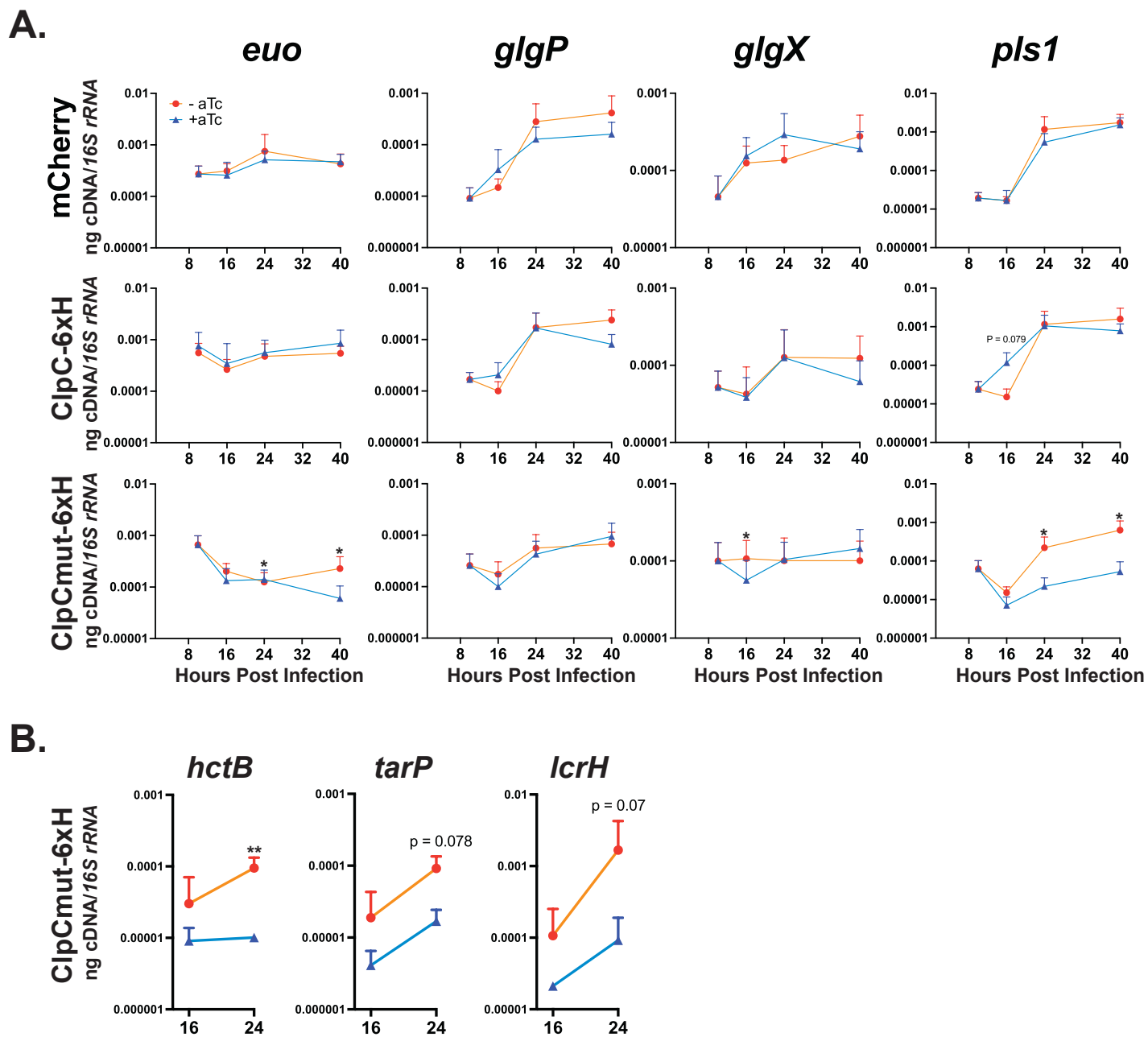

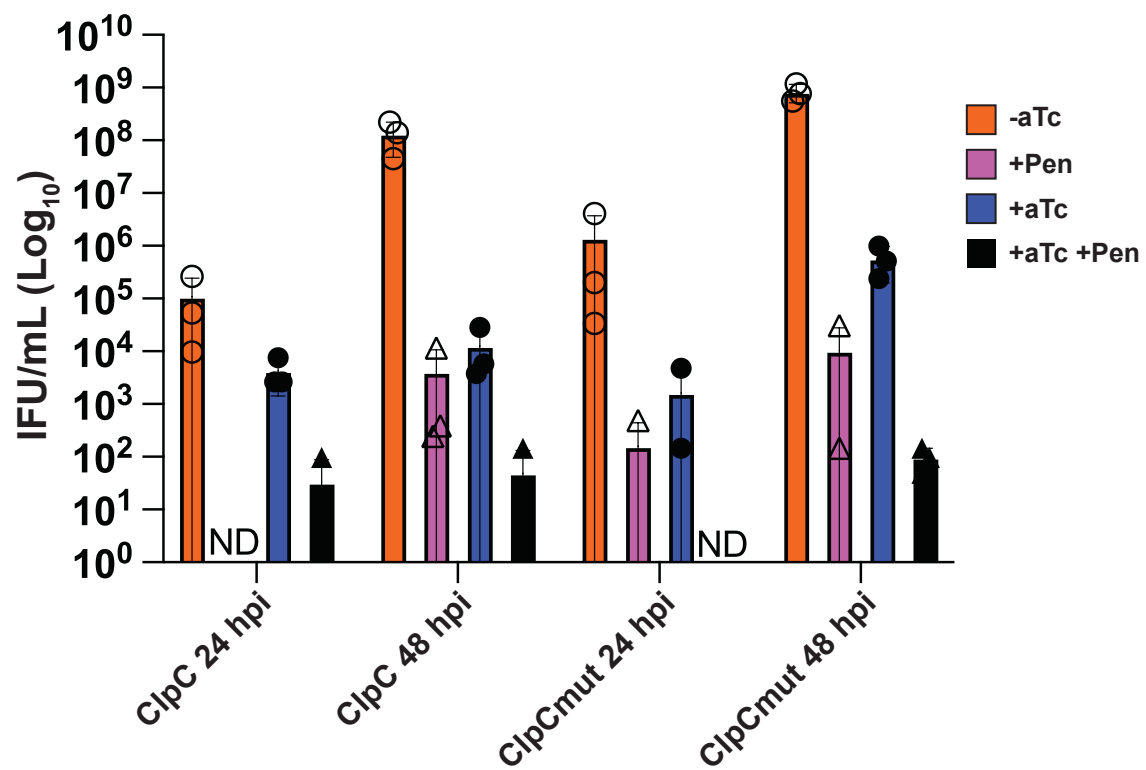

Figure S7

**A.**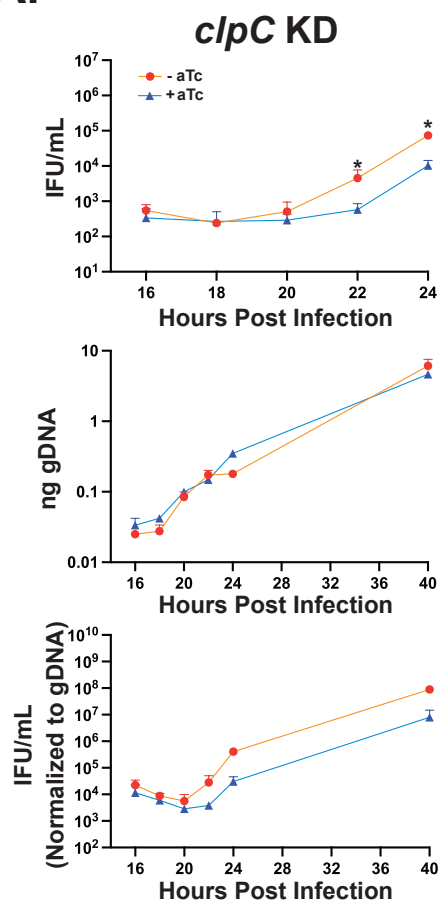**B.**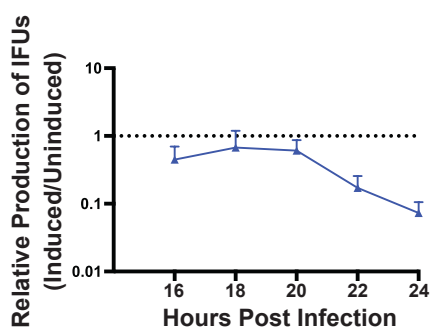**C.**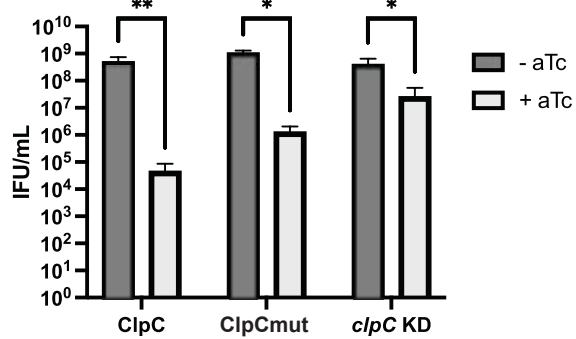

# Figure S8

Supplement: Supplemental Figures — Fig. S1-S8. [file mbio.02870-24-s0001.pdf]
