# Supplementary material for: Overexpressing the ClpC AAA+ unfoldase accelerates developmental cycle progression in Chlamydia trachomatis
Source: mBio. 2024 Nov 22;16(1):e02870-24. doi: 10.1128/mbio.02870-24 (PMC11708050; doi:10.1128/mbio.02870-24)
Supplement: Legends — Supplemental figure legends. [file mbio.02870-24-s0002.docx]

**Supplemental Information**

**Supplemental Figure 1**: **Characterization of pBOMBL_Spc_ Strains.** (A) Effect of overexpressing or not mCherry, ClpC_6xH, or ClpCmut_6xH on inclusion morphology by indirect immunofluorescence analysis. HeLa cells were infected with the indicated strains, and expression of the constructs was induced or not at 10 hpi. At 24 hpi the mCherry infected cells were fixed with 3.25% formaldehyde and 0.125% glutaraldehyde then permeabilized with MeOH to retain mCherry fluorescence (visualized in the green channel). HeLa cells infected with *Chlamydia* expressing ClpC_6xH or ClpCmut_6xH were fixed with MeOH. Fixed cells were stained for major outer membrane protein (red for MOMP) or 6xH (green for ClpC_6xH and ClpCmut_6xH tagged protein from plasmid induction), and DNA (light blue). Representative images were taken in triplicate on a Zeiss Apotome at ×100 magnification with ×5.5 digital zoom. Scale bar = 2 µm. (B) Quantification of recovered inclusion forming units (IFUs) of each transformant from (A). Samples were collected at 24 hpi in triplicate and combined, frozen at -80°C, and titrated onto a fresh monolayer of HeLa cells to quantify inclusions in the secondary infection. GFP-positive inclusions were counted in the secondary infection as a marker for viable EBs (IFU/mL). Statistical significance analysis was performed via a parametric, ratio Student’s T-test (*p<0.05; ****p<0.0001). Data are the average of at least three independent experiments.

**Supplemental Figure 2: Glycogen Accumulates Within the Inclusion Earlier in Development During Overexpression of ClpC_6xH.** Transmission electron microscopy of *C. trachomatis* L2 overexpressing ClpC_6xH from a plasmid encoding penicillin resistance (see Pan, Jensen et al., 2023 [26]). HeLa cells were infected with the indicated strains and, at 10 hpi, expression of the construct was induced or not with anhydrotetracycline (aTc). Samples were processed for TEM at 24 hpi. Inset images were performed on the microscope. Scale bar: 2 μm. See also Main Figure 1 for comparison to spectinomycin-resistant ClpC_6xH overexpression strain.

**Supplemental Figure 3**: **Validation of *glgA* and *glgP* Knockout Strains.** PCR or western blot confirmation of Targetron insertion for *glgA* (A, B) and *glgP* (C, D). By PCR, with the Targetron insertion, gene size should increase ~1.8 kbp from the *bla* insertion. Western blot from D is one image where redundant lanes were removed between the ladder and the three lanes shown. L: Ladder, L2: wild-type L2, L2R: wild-type L2 lacking endogenous pL2 plasmid (Note: glycogen biosynthesis requires the L2 plasmid since the plasmid gene protein Pgp4 activates *glgA* transcription), MOMP: major outer membrane protein. (E) Indirect immunofluorescence assay (IFA) and Periodic Acid Schiff (PAS) stain of CtrL2, CtrL2∆glgA, and CtrL2∆glgP infected cells. Notice how the *glgA* and *glgP* knockout strains are not fluorescent by their respective antibodies, and *glgA* does not produce positive staining by iodine or PAS staining. Scalebar for IFA = ~5 μm. Scalebar for PAS = ~50 μm.

**Supplemental Figure 4: Knockout of *glgP* Increases the Accumulation of Glycogen Within the Inclusion.** Periodic-acid Schiff staining of HeLa cells infected with a *glgP* knockout *Chlamydia* overexpressing ClpC_6xH or ClpCmut_6xH. Expression of the constructs was induced or not at 10 hpi then fixed with MeOH at 27 hpi (N = 3) or 47 hpi (N = 2). Representative images were taken on a Nikon Eclipse Ti-E microscope using a 100× oil objective. Green = 6xH, magenta = Schiff reagent. Arrows point to inclusions. Scale bar: 10 μm.

**Supplemental Figure 5**: **Quantification of Ribosomal RNA Levels, Genomes, and Plasmids of pBOMBL_Spc_ Strains.** qPCR of cDNA or gDNA collected from HeLa cells infected with the indicated strains. Expression of the indicated constructs was induced or not at 10 hpi, and samples were collected at 10, 16, 24, and 40 hpi for RNA and DNA processing. Plasmid per bacteria was calculated by dividing the pDNA value by the gDNA value. Statistical significance analysis was performed via a parametric, ratio Student’s T-test (*p<0.05; **p<0.01). Data are the average of at least three independent experiments.

**Supplemental Figure 6**: **RT-qPCR Analysis of Selected Genes from *C. trachomatis* L2 Overexpressing the Indicated Constructs.** (A) HeLa cells were infected with *C. trachomatis* L2 strains carrying plasmids to inducibly express mCherry, ClpC_6xH, or ClpCmut_6xH. At 10 hpi, expression of the constructs was induced or not with anhydrotetracycline (aTc). RNA was collected at 10, 16, 24, and 40 hpi then processed for RT-qPCR where cDNA was measured for an early gene *euo*, glycogen associated genes *glgP* and *glgX*, and the Pgp4 regulated gene *pls1*. (B) ClpCmut_6xH with the same conditions as (A) but collected at 16 and 24 hpi only. Genes shown are known late genes *hctB*, *tarP*, and *lcrH_1* (*scc2*). cDNA is normalized to *16S rRNA*. Statistical significance analysis was performed via a parametric ratio Student’s T-test (*p<0.05; **p<0.01). Data are the average of at least three independent experiments.

**Supplemental Figure 7**: **Overexpression of ClpC Isoforms When the Developmental Cycle Is Blocked Is Not Sufficient to Generate Infectious Progeny.** Quantification of recovered inclusion forming units (IFUs) from ClpC_6xH or ClpCmut_6xH expression strains. Strains were untreated (orange bar), treated with Penicillin (Pen; magenta bar), induced with aTc (blue bar), or treated with Pen and induced with aTc (black bar) at 10 hpi. Samples were collected at 24 or 48 hpi in triplicate and combined, frozen at -80°C, and titrated onto a fresh monolayer of HeLa cells to quantify inclusions in the secondary infection. GFP-positive inclusions were counted in the secondary infection to quantify viable EBs (IFU/mL). Data are the average of at least three independent experiments. See also Main Figure 3.

**Supplemental Figure 8**: **Knockdown of *clpC* Delays IFU Production.** (A) One-step growth curve of *C. trachomatis* L2 transformed with an anhydrotetracycline (aTc)-inducible dCas12 targeting the *clpC* gene. HeLa cells were infected with this strain, then induced or not at 10 hpi to promote knockdown of *clpC*. Cells were collected between 16-24 and 40 hpi for secondary infection to determine the amount of infectious organisms (IFU/mL), or gDNA for total bacteria. GFP positive inclusions were counted in the secondary infection to assess IFU/mL. (B) IFU/mL normalized to gDNA from (A) was used as a proxy for percent IFUs to total bacteria, thus comparing the amount of IFUs between the induced and uninduced conditions. (C) IFU/mL of overexpression or knockdown strains from Figure 4 or part (A) at 40 hpi. Statistical significance analysis was performed via a parametric, ratio Student’s T-test (*p<0.05; **p<0.01). See also main Figure 4 for more details. Data are the average of at least three independent experiments.

**Supplemental Tables S1-S8. RNA sequencing results from ClpC_6xH overexpression and List of Plasmids, Strains, and Primers used in the study.**
